# Knockdown of L1CAM significantly reduces metastasis in a xenograft model of human melanoma: L1CAM is a potential target for anti-melanoma therapy

**DOI:** 10.1371/journal.pone.0192525

**Published:** 2018-02-12

**Authors:** Ann-Kathrin Ernst, Annika Putscher, Timur R. Samatov, Anna Suling, Vladimir V. Galatenko, Maxim Yu Shkurnikov, Evgeny N. Knyazev, Alexander G. Tonevitsky, Thomas Haalck, Tobias Lange, Hanna Maar, Jennifer Schröder- Schwarz, Kristoffer Riecken, Udo Schumacher, Daniel Wicklein

**Affiliations:** 1 Institute of Anatomy and Experimental Morphology, University Cancer Center, University Medical-Center Hamburg-Eppendorf, Hamburg, Germany; 2 SRC Bioclinicum, Moscow, Russia; 3 Moscow State University of Mechanical Engineering, Moscow, Russia; 4 Department of Medical Biometry and Epidemiology, University Medical Center Hamburg-Eppendorf, Hamburg, Germany; 5 Moscow State University, Moscow, Russia; 6 National Research University Higher School of Economics, Moscow, Russia; 7 Department of Translational Oncology, National Center of Medical Radiological Research, Obninsk, Russia; 8 Outpatient Center, Department of Dermatology, University Medical-Center Hamburg-Eppendorf, Hamburg, Germany; 9 Research Department Cell and Gene Therapy, Department of Stem Cell Transplantation, University Medical Centre Hamburg-Eppendorf, Hamburg, Germany; Istituto Superiore di Sanità, ITALY

## Abstract

Finding additional functional targets for combination therapy could improve the outcome for melanoma patients. In a spontaneous metastasis xenograft model of human melanoma a shRNA mediated knockdown of L1CAM more than sevenfold reduced the number of lung metastases after the induction of subcutaneous tumors for two human melanoma cell lines (MeWo, MV3). Whole genome expression arrays of the initially L1CAM high MeWo subcutaneous tumors revealed unchanged or downregulated genes involved in epithelial to mesenchymal transition (EMT) except an upregulation of Jagged 1, indicating a compensatory change in Notch signaling especially as Jagged 1 expression showed an increase in MeWo L1CAM metastases and Jagged 1 was expressed in metastases of the initially L1CAM low MV3 cells as well. Expression of 17 genes showed concordant regulation for L1CAM knockdown tumors of both cell lines. The changes in gene expression indicated changes in the EMT network of the melanoma cells and an increase in p53/p21 and p38 activity contributing to the reduced metastatic potential of the L1CAM knockdowns. Taken together, these data make L1CAM a highly interesting therapeutic target to prevent further metastatic spread in melanoma patients.

## Introduction

Malignant melanoma is an extremely dangerous disease with high mortality rates due to the aggressive metastatic potential of melanoma cells. Although the development of new therapies for patients with already metastasized melanoma over the last few years resulted in prolonged survival, for a considerable number of patients these new therapies still do not achieve stable remission for more than a few months (see [1] for current review). For example, treatment with agents directed against mutated BRAF alone eliminates visible metastases shortly after first administration; however, due to resistance development, metastatic disease reoccurs after 6–8 months [2, 3]. Additionally, BRAF therapy is contraindicated for patients with BRAF wildtype melanoma und thus this treatment is not feasible for roughly half of the patients. Current ideas for better disease control include a combination of treatments. In cases with BRAF mutated melanoma, combining BRAF and MEK inhibitors further delays the development of resistance to about 11 months and patients with metastases at fewer than 3 organ sites and low LDH can even be stabilized for years [2]. Finding additional therapeutic targets on melanoma cells, preferably molecules which play a functional role in metastasis could greatly enhance chances for developing such combination therapies.

Originally described to play a major role in nervous system development [4], the immunoglobulin superfamily cell adhesion molecule L1CAM was later reported to be involved in cancer progression and metastasis [5] (see [6] for a current review on all aspects of L1CAM). L1CAM expression is of prognostic value in several cancer entities [7] and is thus considered as a promising target for therapy [8]. As L1CAM expression is associated with metastasis in melanoma patients [9], it could be a target for the combination therapies described above. However, until now it has never been demonstrated that L1CAM has a direct functional role in melanoma metastasis, e.g. by an animal model. Available studies focused on primary tumor development [10] or were *in vitro* [11]. Recently, L1CAM has been suggested to be a key mediator of the pro-metastatic role played by core fucosylation by FUT8 in melanoma [12]. Aim of this study was to investigate whether L1CAM plays this functional role in melanoma metastasis in a model comprising the whole metastatic cascade. For this purpose, we used a spontaneous metastasis xenograft mouse model of human BRAF wildtype melanoma cells with and without RNAi mediated L1CAM knockdown. To our knowledge such a spontaneous metastasis xenograft model (comprising the whole metastatic cascade) on the role of L1CAM had never been carried out for any cancer entity before. Using this model, we could demonstrate a functional role in melanoma metastasis for L1CAM as its knockdown resulted in significantly reduced spontaneous metastasis and also in a reduction of EMT-related gene expression and increased p53/p21 and p38 activity in the xenograft tumors.

## Material and methods

### Cell culture

MeWo and MV3 cells were obtained and cultivated as previously described [13]. Both cell lines were certified by the DSMZ (Leipniz Institute DSMAZ-German Collection of MIcroorganims and Cell Cultures). Cell culture medium for selection and cultivation of transfected cells was additionally supplemented with 0.5 μg / ml puromycin (Life Technologies). Human umbilical vein endothelial cells (HUVEC) were obtained from PromoCell (Heidelberg, Germany) and were cultivated in Endothelial Cell Growth Medium and Supplement according to the manufacturer’s instructions.

### RNAi mediated knockdown of L1CAM

L1CAM knockdown in MeWo and MV3 cells (MeWo and MV3 L1 kd) was achieved by an shRNA mediated approach: A 65 bp DNA oligomer containing a 19 bp anti-L1CAM sequence (GGATGGTGTCCACTTCAAA) [14] was inserted into the pLVX vector (Clontech, Saint-Germain-en-Laye, France) according to the manufacturer’s instructions. The sequence was checked for potential off-target effects using NCBI BLAST (plus/plus and plus/minus strands down to an E value of 170) and found none of these off-targets regulated (i.e. none can be found in the S1 and S2 Tables). The same vector containing a sequence against firefly luciferase was used to generate a transfected control cell line (MeWo and MV3 Luc). Viral particles were produced as cell-free supernatants by transient transfection of HEK-293T packaging cells as described [15]. In brief, lentiviral vectors based on pLVX-shRNA1 were packaged using the second-generation packaging plasmid psPAX2 (Addgene #12260, Addgene, Teddington, UK) and phCMV-VSV-G [16] expressing the envelope protein of vesicular stomatitis virus. The supernatant was harvested 24 hours after transfection, 0.45 μm filtered and stored at -80°C. Target cells were plated at 5 × 10^4^ cells in 0.5 ml medium in each well of a 24 well plate. After addition of viral particle containing supernatant to the cells, the medium was replaced the next day and puromycin was added the second day after transduction at a concentration of 1 μg / ml. The puromycin selection was carried out for at least one week. Melanoma cells L1CAM kd and Luc were seeded at 3 cells per well in two 96 well plates each and growing MeWo and MV3 sublines were screened for L1CAM expression using flow cytometry. Sublines with normal (compared with the original cell lines, Luc) and low (>85% reduced fluorescence signal, L1 kd) L1CAM were pooled and used for further experiments.

### Proliferation, invasion, colony forming and laminar flow adhesion *in vitro* assays

The Cell Proliferation Kit II (XTT, Roche Diagnostics, Mannheim, Germany) was used for *in vitro* proliferation assays according to the manufacturer’s instructions with 5 X 10^4^ cells seeded per well and 48 h proliferation time.

Invasive potential was tested *in vivo* using the 8 μm pore size, 24-multiwell BioCoat System (BD) with Calcein AM (eBioscience, Frankfurt, Germany), according to the manufacturer’s instructions. 1 X 10^5^ L1 kd or Luc cells were seeded per well and incubated for 24 h. Fluorescence signals were analyzed with a Genios Reader (Tecan, Männerdorf, Switzerland).

L1 kd pool or Luc cells (200 / ml) were resuspended in a 1:1 mixture of growth factor reduced matrigel (BD) and complete RPMI1640. 50 μl of the cell suspension were deposited in each well of a 96-well plate. 200 μl complete RPMI were then added to each well and changed twice weekly. Colonies were counted and evaluated using a light microscope after 14 days. Adhesive interactions of melanoma cells with HUVEC were analyzed under physiological shear stress conditions in ibidiTreat μ-slide IV^0.4^ flow chambers with or without pre-incubation of HUVEC with 10ng/μl rhTNFα for 4h as described (Richter et al., 2011, Lange et al., 2014). Adhesive events were digitally recorded in three regions of interest per flow chamber and the number of events per minute was subsequently analyzed using CapImage Software (Dr. Zeintl, Heidelberg, Germany).

### Flow cytometry

Flow cytometry was performed as described [17]. Briefly, cells were stained with phycoerythrin-labeled mouse anti-human L1CAM (anti-CD171, clone 5G3, eBioscience) or the corresponding isotype control (murine IgG2a-PE, eBioscience). Stained cells were subjected to fluorescence assisted flow cytometry on a FACSCalibur (BD). Files were analyzed using Win MDI 2.9 software.

### Xenograft mouse model of human melanoma

The experiment was carried out as previously described [13]. Briefly, 10 (MV3 Luc and L1CAM kd) and 10 (MeWo Luc and L1CAM kd) scid mice in each group received subcutaneous injections of 10^6^ melanoma cells with or without L1CAM1 knockdown, respectively. Mice with ulcerated tumors or tumors with an estimated mass exceeding 10% of the respective animal’s weight were euthanized immediately. 3 and 4 animals were lost to murine lymphoma in the L1 kd and Luc group, respectively. Thus, this part of the experiment was repeated with 15 mice per group. This time, 1 and 2 animals were lost to murine lymphoma in the L1 kd and Luc group. In the MV3 group, only one mouse from each group developed lymphoma. Taken together, data from 18 (MeWo Luc), 21 (MeWo L1 kd), 9 (MV3 Luc) and 9 (MV3 L1 kd) animals were included in the further analyses.

The methodology for carrying out the animal experiments was consistent with the UKCCR guidelines for the welfare of animals in experimental neoplasia [18]. The experiment was recommended and supervised by the institutional animal welfare officer and approved by the local licensing authority (Behörde für Soziales, Familie, Gesundheit, Verbraucherschutz; Amt für Gesundheit und Verbraucherschutz; Billstr. 80, D-20539 Hamburg, Germany) under the project no. G10/55.

All animals used were pathogen-free Balb/c severe combined immunodeficient (scid) mice aged 9–14 weeks with a weight of 25–30 g at the beginning of the experiments. The mice were housed in filter-top cages, provided food and water ad libitum and their condition was monitored daily. Apart from visible tumors, the general condition of the animals was evaluated by a standardized in house scoring system based on movement/behavior, weight development, food and water intake and fur condition. The mice were killed by cervical dislocation after having been anesthetized by intraperitoneal injection of a weight-adapted dose (10 μl / g bodyweight) of a mixture of 1.2 ml Ketamin (Gräub AG, Bern, Switzerland), 0.8 ml Rompun (Bayer AG, Leverkusen, Germany) and 8 ml saline.

### Immunohistochemistry

Immunohistochemical staining for L1CAM using sections of paraffin-embedded tissues was carried out as previously described [13] [19]. To increase staining intensity, L1CAM staining for the inintially L1CAM low melanoma cells MV3 was modified in that the K5005 AP/RED Rabbit/Mouse staining kit (Dako, Copenhagen, Denmark) was used after the primary antibody. Other stainings were carried out as follows: Notch 1 (Abcam, Cambridge, UK; cat. no. ab52627; Clone EP1238Y) at 19.2 μg / ml; pretreatment steamer at 121°C for 10 min; citrate buffer pH 6. Jagged 1 (Jag1) (Sigma-Aldrich, St. Louis, MO, USA; cat. No. HPA021555) at 6 μg / ml; water bath at 99°C for 20 min; S1699 retrieval solution pH 6 (Dako). p53 (Dako; cat.no. M7001; clone DO-7) at 1.37 μg / ml, microwave 2X 4 min at 800 W, citrate buffer pH 6. p21 Thermo-Fisher, Waltham, MA, USA; cat.no. MS-891-P; clone CP74400) at 1 μg / ml; steamer at 121°C for 10 min; S1699 pH6. p38 (Abcam; cat.no. ab38238; clone pohosoha T180 + Y1820) at 2 μg / ml, steamer at 100°C for 20 min, S1699 pH6 with K5005 AP/RED treatment after primary antibody.

Stained slides were scanned by a Mirax microscope (Zeiss, Jena, Germany) and the Panoramic Viewer software (3D Histech, Budapest, Hungary) was used to take images.

### Quantification of lung metastasis

Lung metastases were quantified as previously described [20]. Briefly, whole paraffin-embedded lungs were cut into serial slices, every tenth slice was HE stained, the number of metastasis counted in 10 slices and total lung metastases were calculated for each animal based on the respective total number of slices.

### Nucleic acid extraction and real-time PCR

DNA and RNA extraction from murine blood and tumors as well as quantification of human melanoma cells by quantitative real-time polymerase chain reaction (qRT-PCR) for human *Alu* sequences was performed as previously described [19].

### Statistics

Continuous variables are displayed using boxplots.

In all models the interaction of cell line (MeWo vs. MV3) and group (Luc vs. L1 kd) was tested using a likelihood-ratio test. In case of a significant interaction the group comparison was presented separately for each cell line and in case of insignificance the interaction was excluded from the model and an overall group comparison was presented.

*In vitro* outcomes (proliferation, invasion, colony forming assay and laminar flow) were analyzed using linear models. Outcomes of the xenograft model were survival, tumor weight, metastases, and number of circulating tumor cells (CTCs; at the time of death in the animals’ blood, determined by qRT-PCR). Survival of mice was defined as time until mice were euthanized when the tumor ulcerated or reached an estimated mass exceeding 10% of the respective animal’s weight. As no censoring was existent it was analyzed using a linear model which was moreover adjusted for the type of death (ulcerated vs. not ulcerated). Tumor weight was analyzed in an analogous model. Number of metastases was analyzed and evaluated/quantified using a negative binomial regression which was adjusted for survival time and tumor weight and included furthermore CTCs as interesting variable. CTCs as outcome variable were logarithmized and analyzed using a linear model, which was adjusted for survival time and tumor weight.Parameter estimates are presented together with 95% confidence intervals (95%-CI). A two-tailed p<0.05 was considered to be statistically significant. All analyses were conducted using Stata 14.1 (StataCorp LP, College Station, Texas, USA).

### Microarray protocol

Total RNA was extracted from the frozen tumor samples using the miRNeasy Mini Kit (Qiagen, Hilden, Germany) as recommended by the manufacturer. RNA concentrations were determined by the Nanodrop 1000 spectrophotometer (Thermo Fisher Scientific, Waltham, MA, USA). RNA quality was checked using the Experion RNA StdSens Analysis Kit and Experion platform (Bio-Rad, Hemel Hempstead, United Kingdom). For all samples RQI value (RNA Quality Indicator) was greater than 8.

Microarray expression analysis was performed using GeneChip® Human Transcriptome Array 2.0 (Affymetrix, High Wycombe, United Kingdom) as recommended by the manufacturer. Data processing was carried out using Signal Space Transformation–Robust Multi-Array Analysis (SST-RMA) method in Affymetrix Expression Console (version 1.4.1.46). Dispersion analysis (ANOVA) was applied to the logarithmic expression values using Affymetrix Transcriptome Analysis Console (version 3.0.0.466). For p-values correction we used the Benjamini-Hochberg method [21]. Analysis of regulation profiles was performed using Pathway Studio software (Elsevier, Amsterdam, Netherlands).

## Results

### Knockdown of L1CAM

The newly generated sublines were analyzed for L1CAM expression by flow cytometry.

**Fig 1:**
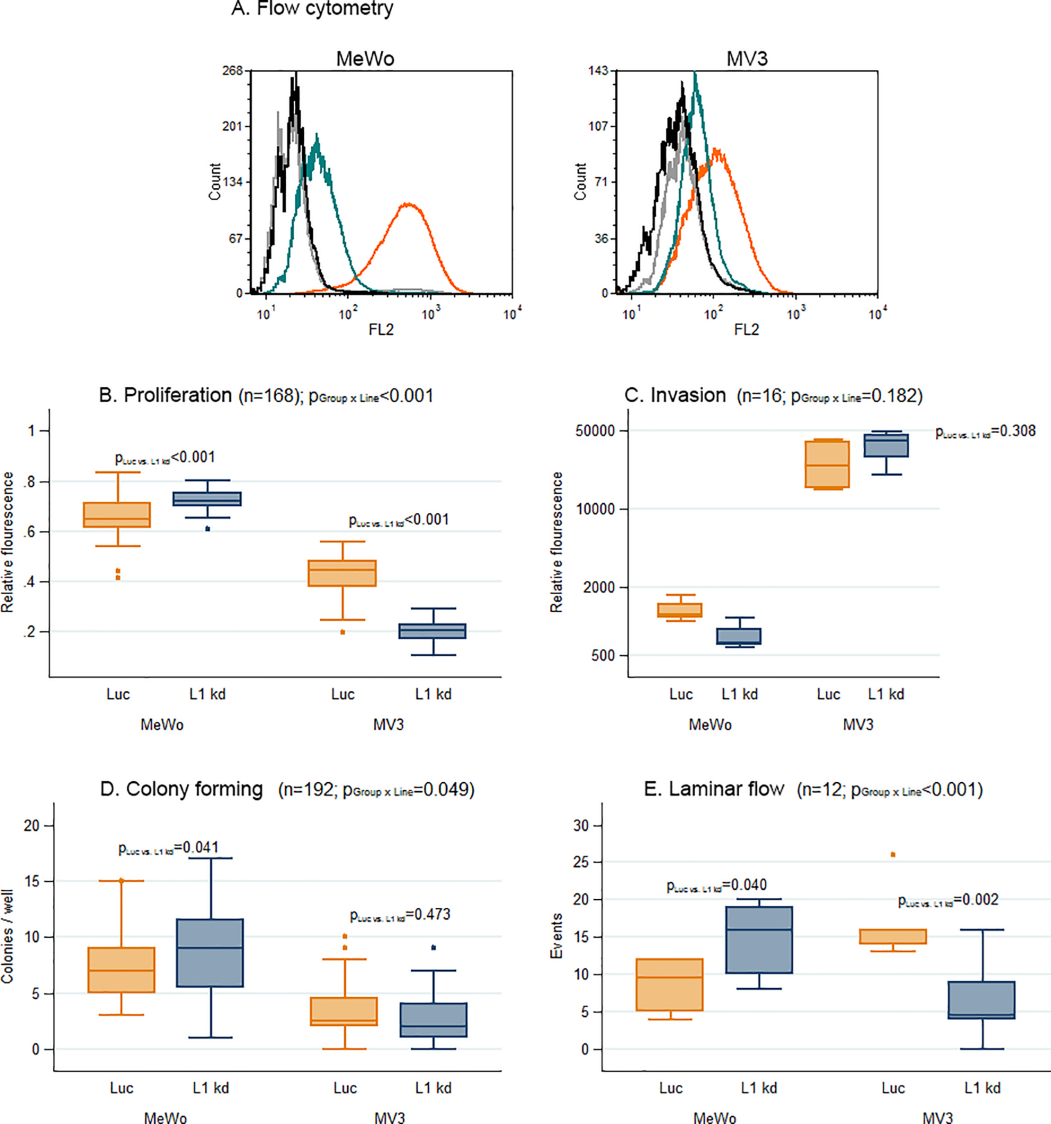
In vitro experiments yielded inconclusive results for predicting in vivo metastatic potential of L1CAM knockdown melanoma cells. Statistics: All numbers n represent the total number of individual samples (each from one individual animal) of the respective experiment. All values of P are derived from linear models. If P_Group X Line_ is below 0.05 (i.e. if L1CAM knockdown displays different effects for cells from the two lines), a separate P_Luc vs. L1kd_ is given for each cell line. Flow Cytometry: Staining of MeWo Luc and L1CAM knockdown (L1 kd) (left panel) and MV3 Luc and L1 kd (right panel) for L1CAM. In contrast to the Luc controls, surface L1CAM1 is reduced by more than 85% (MeWo) and apporoximately 75% (MV3) on the L1 kd cells (A). Proliferation assay: 5 X 10^4^ cells seeded per well and incubation for 48 h: Significant increase in proliferation for MeWo with L1CAM knockdown (L1 kd) compared with the MeWo Luc controls with unchanged surface L1CAM and significant decrease for MV3 L1 kd compared with MV3 Luc (B). Invasion assay: 1 X 10^5^ cells were seeded per well and incubated for 24 h: No significant change in the L1 kd knockdown cells’ ability for invasion (C). Colony forming assay: Cells were seeded in matrigel / culture medium in a 96-well plate; colonies were counted and evaluated using a light microscope after 14 days: Significant increase in colony numbers for MeWo L1 kd cells against MeWo Luc and no significant difference in colony numbers between MV3 L1 kd cells and MV3 Luc controls was observed (n = 48, each) (D). Laminar flow adhesion assay on activated endothelium: Interactions of melanoma cells suspended in culture medium with a HUVEC monolayer under laminar flow. The number of events (only adherence and tethering, no rolling events) increased significantly for MeWo L1 kd against MeWo Luc cells and decreased significantly for MV3 L1 kd against MV3 Luc cells (E).

Sublines with more than 85% (MeWo) and 75% (MV3) reduced L1CAM expression were pooled and used for all further experiments (designated MeWo and MV3 L1 kd). MeWo Luc and MV3 Luc cells with unchanged L1CAM expression, but with puromycin resistance were used as controls (**Fig 1A**).

### *In vitro* proliferation and migration

In an XTT assay, MeWo L1 kd cells showed a significant increase in proliferation (+0.07, 95%-CI [0.04;0.10]; p<0.001) compared with the MeWo Luc controls with unchanged surface L1CAM. For MV3 L1 kd there was a decrease in proliferation (-0.22, 95%-CI [-0.24;-0.19]; p<0.001) in comparison to MV3 Luc controls (**Fig 1B**).

We found no significant overall influence of L1CAM knockdown on the cells’ ability for *in vitro* invasion in a transwell assay (p = 0.308; **Fig 1C**).

In a colony forming assay in matrigel, a significant increase of colony numbers for MeWo L1 kd cells in comparison to Luc controls was observed (+1.25, 95%-CI [0.05;2.45]; p = 0.041) while there was no significant difference in colony numbers between L1 kd cells and Luc controls for MV3 (p = 0.473; **Fig 1D**).

In laminar flow assays measuring adherence, tethering and rolling on activated endothelial cells (HUVEC), the number of events increased significantly for MeWo L1 kd cells (+6.17, 95%-CI [0.32;12.02]; p = 0.040) while it decreased for MV3 L1 kd cells (-10.67, 95%-CI [-16.80;-4.53]; p = 0.002; **Fig 1E**).

Summarized, knockdown of L1CAM *in vitro* displayed different effects (proliferation, colony forming and laminar flow adhesion) or no effect at all (invasion) for both human melanoma cell lines used. These *in vitro* results do not reflect the *in vivo* results from the following xenograft model (see below).

### L1CAM knockdown decreases lung metastasis by the factor 7.26 in a xenograft model of human melanoma

All animals developed subcutaneous tumors at the injection site and were euthanized when the tumor ulcerated or reached an estimated mass exceeding 10% of the respective animal’s weight. The time span between injections of melanoma cells until euthanization will subsequently be referred to as survival time.

**Fig 2:**
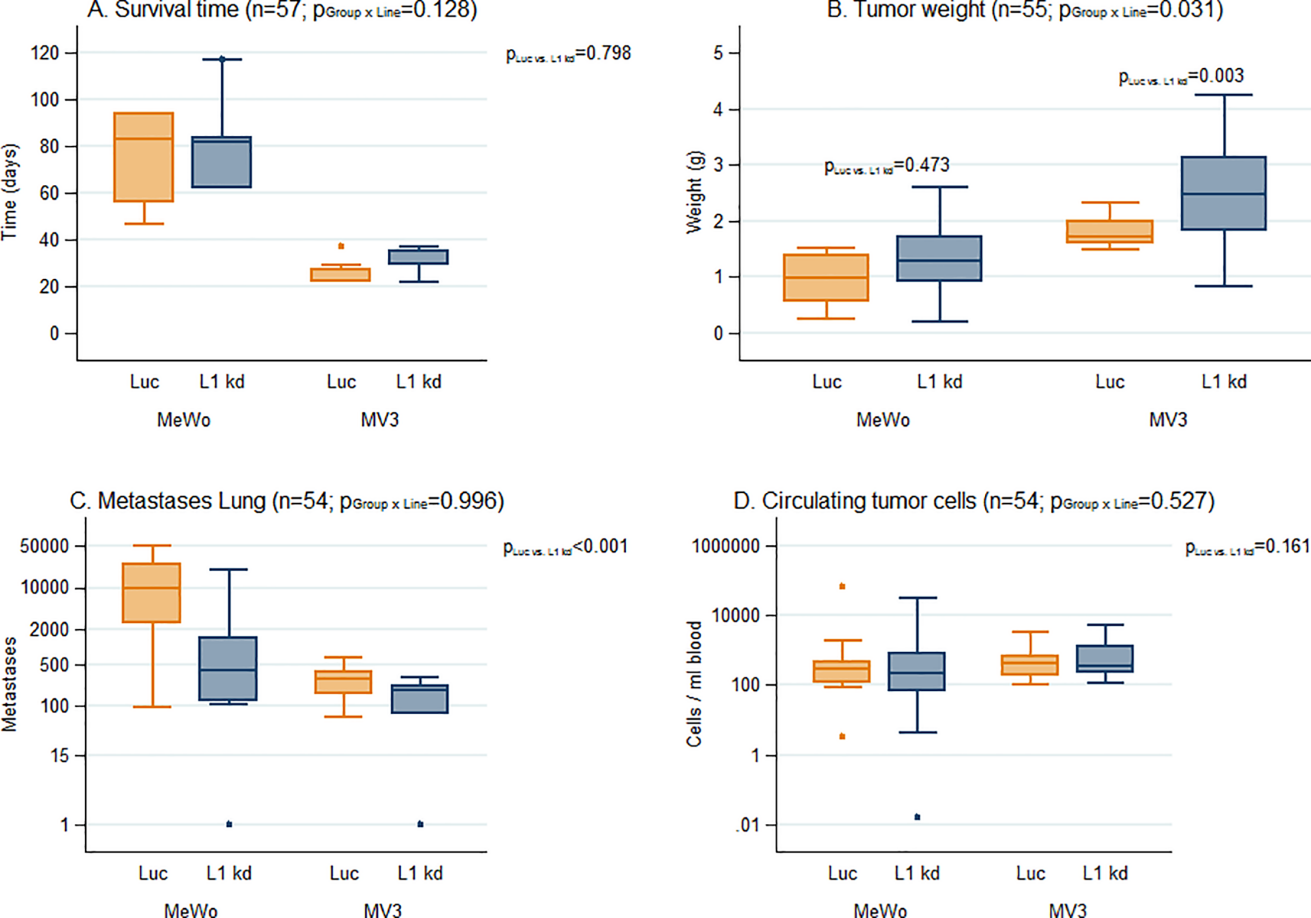
Xenograft spontaneous metastasis model of human melanoma cells with high L1CAM expression (MeWo) in scid mice. **L1CAM knockdown reduces metastasis by more than 80%.** Statistics: All numbers n represent the total number of individual samples of the respective experiment. Survival of mice was analyzed using a linear model which was moreover adjusted for the type of death (ulcerated vs. not ulcerated). Tumor weight was analyzed in an analogous model. Number of metastases was analyzed and evaluated/quantified using a negative binomial regression which was adjusted for survival time and tumor weight and included furthermore CTCs as variable. CTCs as outcome variable were logarithmized and analyzed using a linear model, which was adjusted for survival time and tumor weight. If PGroup X Line is below 0.05 (i.e. if L1CAM knockdown displays different effects for the two cell lines), a separate PLuc vs. L1kd is given for each cell line. Animals’ survival after subcutaneous injection of 10^6^ melanoma cells: L1CAM knockdown did not significantly change the animals’ survival time (A). The weight of the MeWo L1 kd tumors was not significantly different from the MeWo Luc control tumors, the weight of the MV3 L1 kd tumors was significantly higher than the tumor weight of the MV3 Luc control tumors (B). The number of lung metastases was significantly lower (reduced 7.26 fold) in the L1 kd than in the Luc control groups both for MeWo and MV3 (C). The numbers of circulating tumor cells (CTC) in the animals’ blood were not significantly altered by L1CAM knockdown both for MeWo and for MV3 (D).

L1CAM knockdown did not significantly change the animals’ survival time in MeWo and MV3 (p = 0.798; **Fig 2A**).

Within the MV3 cell line the size of the L1CAM knockdown tumors was significantly greater (+0.83g, 95%-CI [0.30; 1.37]; p = 0.003) in comparison to the MV3 control tumors. The size of the MeWo L1CAM knockdown tumors was not significantly different from the control tumors (p = 0.473; **Fig 2B**).

**Fig 3:**
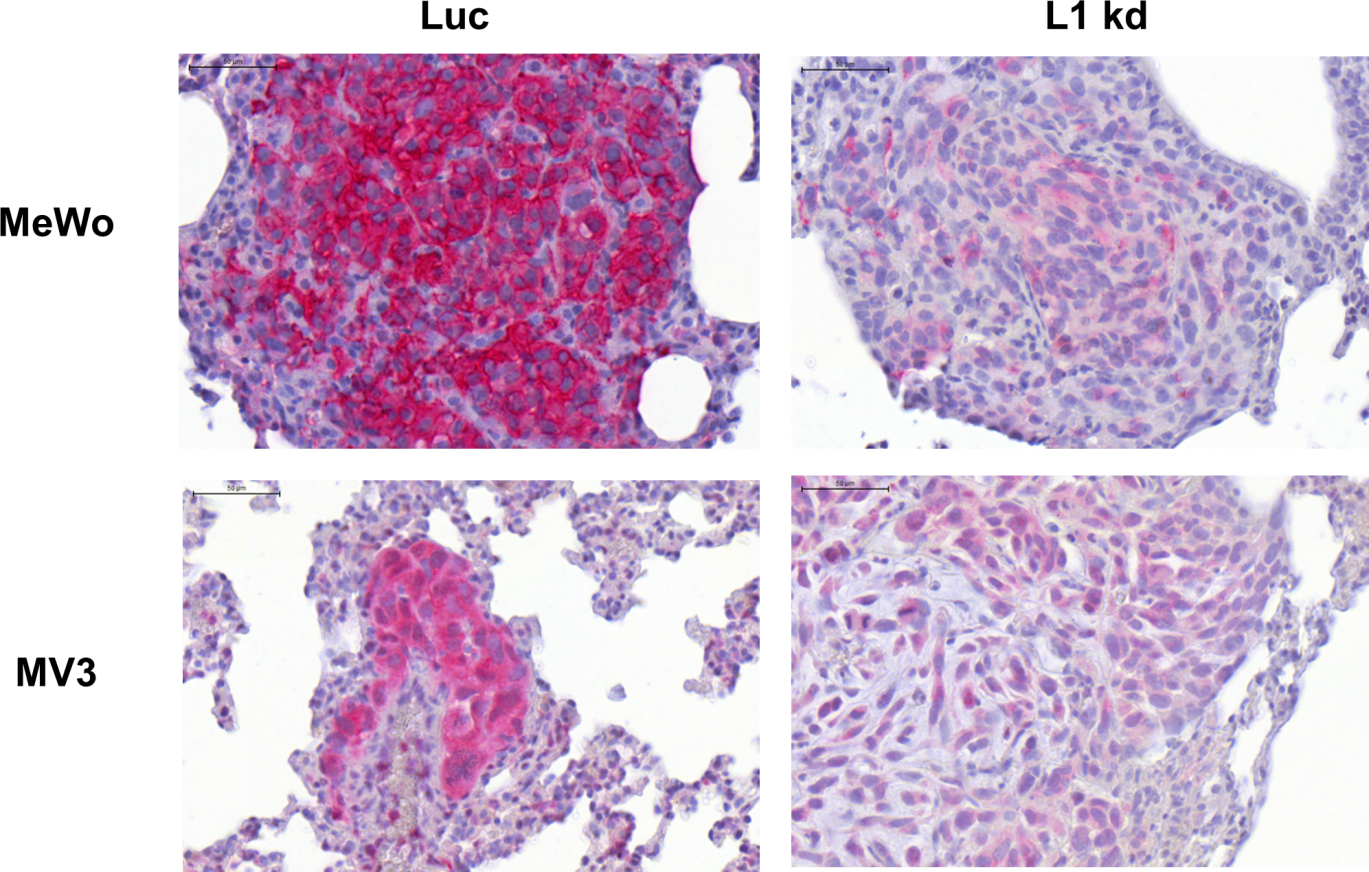
L1CAM expression in lung metastases from human melanoma xenograft as detected by immunohistochemical analysis. Immunohistochemical staining for L1CAM expression (red) in lung metastases of melanoma cells MeWo (upper panels) and MV3 (lower panels) with unchanged L1CAM expression (Luc, right panels) and L1CAM knockdown (L1 kd, left panels). All scale bars: 50 μm. Please note that staining was amplified for MV3.

Metastatic spread of the human melanoma cells was exemplarily studied in the animals’ lungs. All control (Luc) metastases displayed high (MeWo) or low (MV3) L1CAM expression and even most L1 kd metastases still showed weak (MeWo) or very weak (MV3) L1CAM expression (**Fig 3**, please note that staining was intensified for the MV3 samples). Metastasis (adjusted for survival time and tumor weight) was 7.26 times higher in the MeWo and MV3 Luc group in comparison to the L1CAM knockdown group (95%-CI [3.10;17.00]; p<0.001; **Fig 2C**). Survival time also showed a significant influence on lung metastasis. With each day of survival, metastatic spread increased by 6.83% (95%-CI [2.94; 10.86]; p<0.001). Tumor weight and number of CTCs showed no influence on the number of metastases (p = 0.070 and p = 0.436 respectively).

Melanoma cell numbers (determined by RT-qPCR) in the bone marrow of the animals were low: most of the samples showed no values above background, thus only samples with at least 1 melanoma cell per 10^6^ bone marrow cells were considered positive. Despite the low melanoma cells numbers, results for disseminated melanoma cells (DMCs) in the bone marrow show a tendency towards the results for the animals’ lungs: In the MeWo luc group 33.3% of the animals were positve for DMCs, 23.8% were positive in the MeWo L1 kd group. For MV3, 55.6% were positive for DMCs in the Luc group and 22.2% in the L1 kd group.

Regarding influence factors on the number of CTCs we found the mass of the primary tumor to be significant: CTC numbers increased by the factor 3.33 per 1 g increase of the primaries (95%-CI [1.56; 7.09]; p = 0.002). Survival time and L1CAM knockdown showed no significant influence on CTC numbers (p = 0.454 and p = 0.161 respectively) (**Fig 2D**).

Summarized, in a melanoma xenograft model, L1CAM knockdown reduced lung metastasis by the factor 7.26. This reduction is probably not caused by a less aggressively growing primary tumors, a change in survival time or a decrease in CTC number, as all this parameters remain unaltered by L1CAM knockdown in the human melanoma cells.

### Comparison of gene expression of L1CAM knockdown tumors with controls

To gain some insight into how the L1CAM knockdown might cause the drastic reduction in metastasis observed in the xenograft model, we performed a whole (human) genome expression analyses starting with the corresponding primary xenograft tumors of the initially L1CAM high cell line MeWo (MV3 microarray data see below).

**Fig 4:**
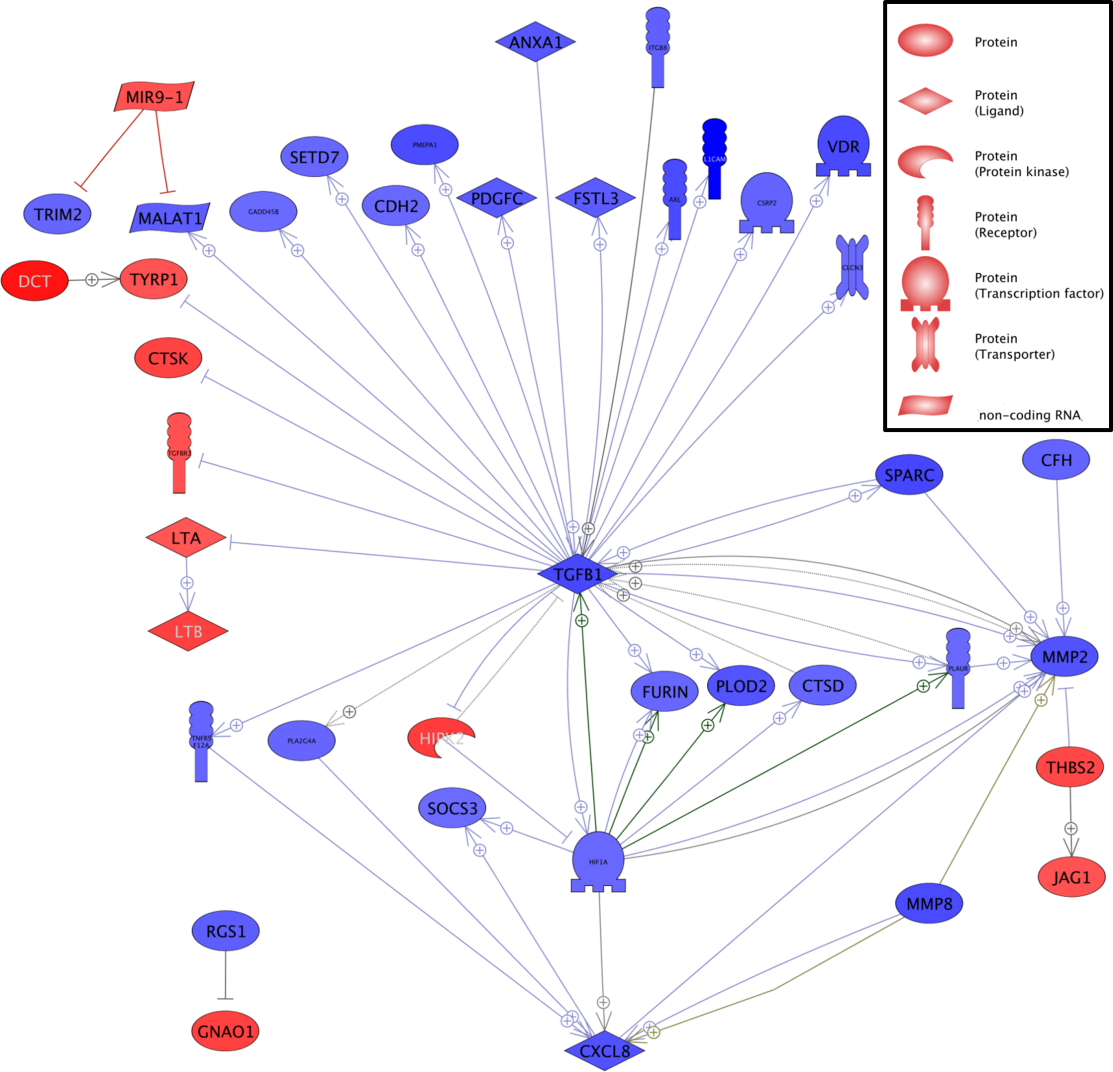
Comparison of the gene expression of subcutaneous MeWo tumors with L1CAM knockdown with expression of MeWo Luc control tumors. **Analysis revealed a central role for TGFβ.** Analysis of the regulation profile in L1CAM knockdown tumors compared with Luc control tumors using the Pathway Studio software including all genes which display a respective 1.5 fold up- or downregulation in expression in the L1 kd tumors. Gene expression upregulated in the L1kd tumors is given in red, downregulated expression in blue.

Considering all genes with a significant (ANOVA p-value and adjusted p-value below 0.05) expression fold change of at least 1.51, in the xenograft tumors formed by the originally L1CAM high MeWo cells, the expression of 981 genes was up- and of 1000 (excluding L1CAM itself) downregulated (**S1 Table**). Analysis of this regulation profile using the Pathway Studio software suggests a central role of Tumor Growth Factor β (TGFB1, which itself shows a 5.06 fold downregulation in the L1CAM knockdown tumors) as many genes that have been reported to be directly or indirectly regulated by TGFβ display a respective up- or downregulation in the L1 kd tumors (**Fig 4**).

As metastasis is often associated with Epithelial to Mesenchymal Transition (EMT) regulated genes were screened for EMT related genes:The phenotype of the L1CAM knockdown tumors appeared less mesenchymal than the control tumors’; e.g. significant reduction of N-cadherin (CDH2), Tyrosine-protein kinase receptor UFO (AXL), Matrix Metalloproteinase 2 (MMP2), TGFB1 and Secreted Protein Rich in Cysteine (SPARC) expression. An exception from this reduction of EMT related gene expression, however, was an increased expression of the NOTCH ligand JAG1 (Jagged 1) in the L1CAM knockdown tumors. As this gene was the sole exception among the EMT related genes, we chose JAG1 to exemplarily verify the array results. Interestingly, the increase in JAG1 expression was accompanied by a significant increase in NOTCH1 and 2 expressions, albeit only 1.40 and 1.41 fold, respectively, and a significant increase in NOTCH signaling activator Contactin 1 (CNTN1) expression. With CCNC (Cyclin-C), HIF1A (Hypoxia-inducible factor 1-α) and KAT2B (K acetyltransferase 2B) three other genes involved in NOTCH signaling are downregulated.

**Fig 5:**
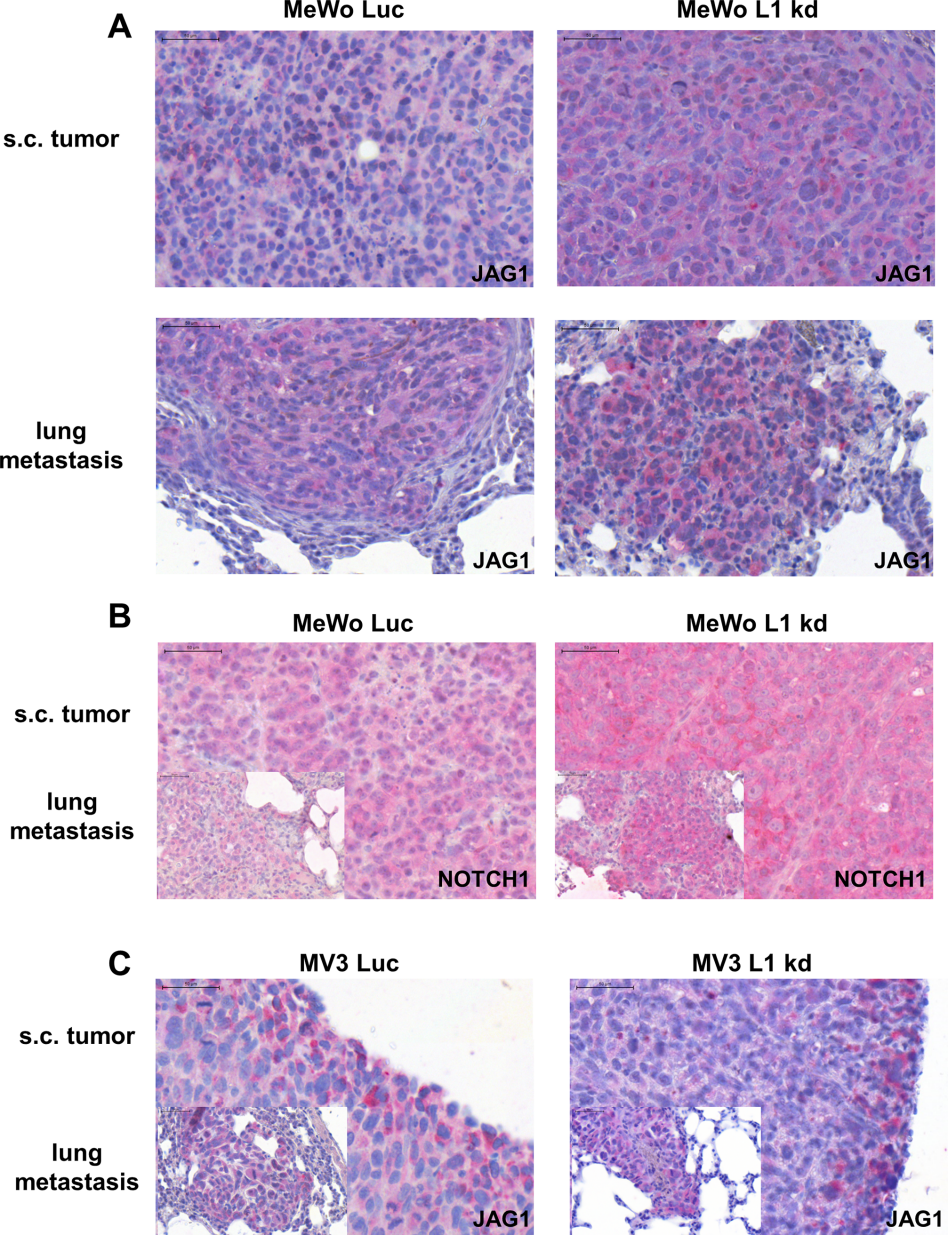
Verification of gene expression array data by immunohistochemical analysis of Jagged 1 and Notch 1 expression in subcutaneous tumors and lung metastases from a human melanoma (MeWo) xenograft experiment. Immunohistochemical staining for Jagged 1 (JAG1) expression (red) in subcutaneous (s.c.) tumors (upper left and upper right panel) and lung metastases (lower left and lower right panel) of melanoma cells (MeWo) with unchanged L1CAM expression (Luc, upper and lower left panels) and L1CAM knockdown (L1 kd, upper and lower right panels). All scale bars: 50 μm. Stainings show a tendency towards increased Jagged 1 for L1 kd tumors and metastases (A). Immunohistochemical staining for Notch1 expression (red) in subcutaneous tumors (upper left and upper right panel) and lung metastases (lower left and lower right panel) of melanoma cells (MeWo) with unchanged L1CAM expression (Luc, upper and lower left panels) and L1CAM knockdown (L1 kd, upper and lower right panels). All scale bars: 50 μm. Stainings show a tendency towards increased Notch 1 for L1 kd tumors and metastases (B). Immunohistochemical staining for Jagged 1 (JAG1) expression (red) in subcutaneous tumors (big panel) and lung metastases (smaller panel, lower left) of MV3 melanoma cells with unchanged L1CAM expression (Luc, left panels) and L1CAM knockdown (L1 kd, right panels). All scale bars: 50 μm. Stainings show no tendency towards altered Jagged 1 for L1 kd tumors and metastases (C).

On protein level, a tendency towards increased Jagged 1 (Fig 4A) and Notch 1 (Fig 4B) expression in the L1 kd tumors could be verified via immunohistochemistry. Additionally, Notch 1 and Jagged 1 expression could be demonstrated in lung metastases with a higher Jagged 1 expression in the few L1 kd metastases (**Fig 5A, Fig 5B**).

**Fig 6:**
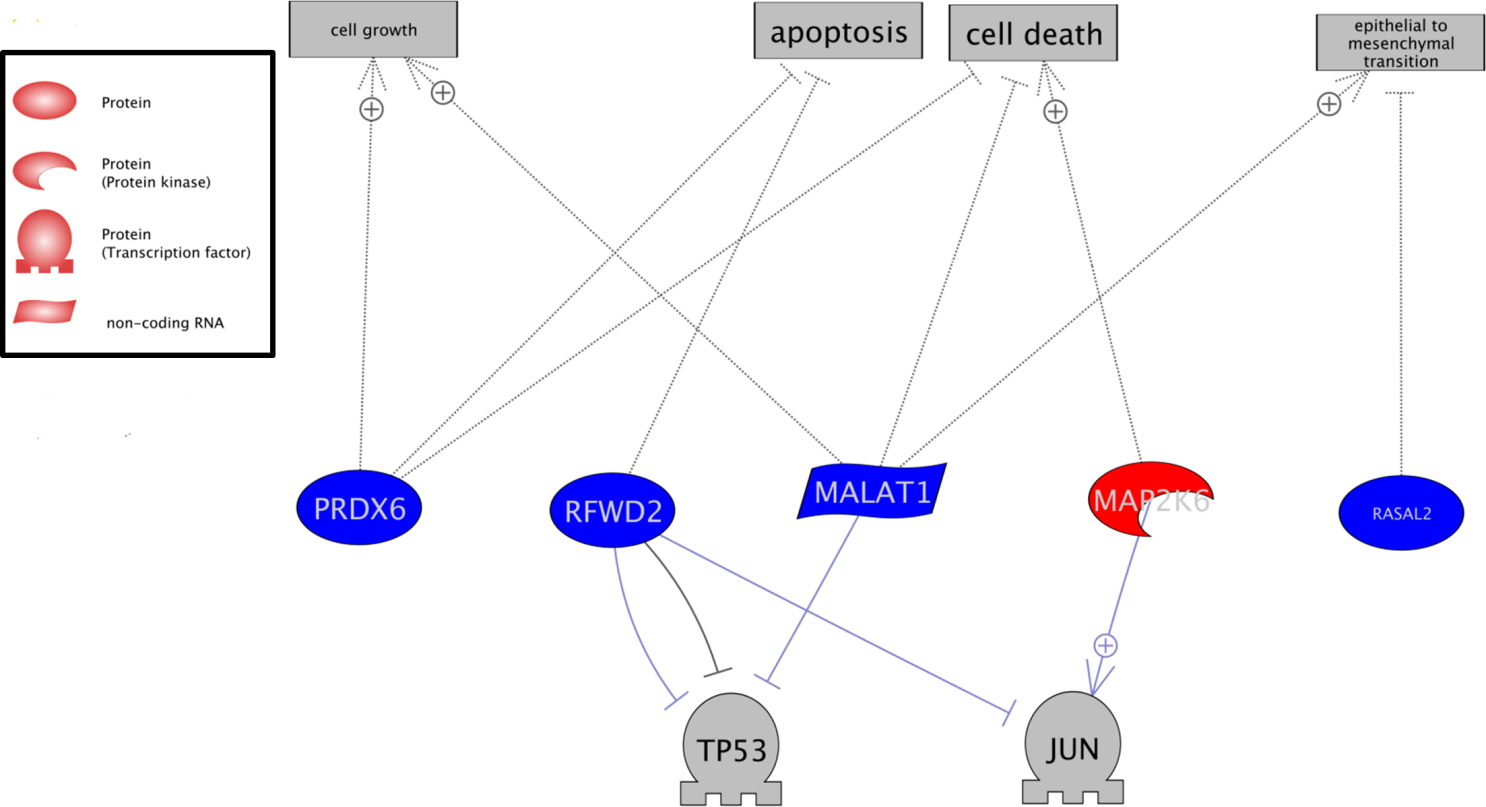
Gene expression with the same regulation (up or down respectively) in MeWo and MV3 subcutaneous tumors with L1CAM knockdown compared with their respective Luc control counterparts with unaltered L1CAM expression. Analysis using Pathway Studio software: Gene expression upregulated in L1CAM knockdown tumors compared with Luc control tumors with unaltered L1CAM; upregulated expression is given in red, downregulated expression in blue, potentially affected factors / processes in grey.

As L1CAM knockdown also resulted in a reduction in metastasis for the initially L1CAM low MV3 cells, an additional whole genome expression analysis of the corresponding MV3 primary xenograft tumors was performed (S2 Table). Genes with concordant regulation (up or down, respectively) for both groups of xenograft tumors (MeWo and MV3) were determined (adjusted p-value below 0.05 and at least 1.5 fold regulation): Excluding L1CAM, 17 genes were found to be regulated in both L1 kd groups (all genes see **Table 1**, analysis see **Fig 6**). Immunohistochemical stainings showed clear Jagged 1 (**Fig 5C**) and Notch 1 expression in the subcutaneous MV3 tumors and lung metastases, however, we did not see a tendency towards higher or lower expression in the L1 kd tumors or metastasis compared with the Luc controls which already displayed a higher Jagged 1 expression than the MeWo Luc tumors (**Fig 5A**). This lack of difference for MV3 is in line with the results on RNA level as no significant difference in Jagged 1 and Notch was detected by analysis of the arrays as well.

**Table 1:** Genes with the same regulation (up or down respectively) in MV3 and MeWo subcutaneous tumors with L1CAM knockdown (L1 kd) compared with their respective Luc control counterparts with unaltered L1CAM (Luc) expression. Included are only fold changes at least +/- 1.5; ANOVA and adjusted p-values <0.05, both for MV3 and MeWo tumors.

Gene symbol	MV3expressionlinear foldchangeL1 kd vs.Luc	MeWoexpressionlinear foldchangeL1 kd vs.Luc
MALAT1	-5.77	-2.42
RASAL2	-2.62	-2.88
TOR1AIP2	-2.28	-1.75
L1CAM	-2.20	-96.76
PRDX6	-2.20	-1.59
KCNQ1OT1	-2.19	-1.54
XPR1	-2.12	-1.82
DARS2	-2.09	-1.50
CACYBP	-2.01	-2.16
KIFAP3	-1.97	-1.78
SOAT1	-1.96	-1.52
TSEN15	-1.84	-1.79
NME7	-1.79	-1.50
ARID5B	-1.76	-1.54
RFWD2	-1.69	-1.67
ZNF432	-1.66	-1.52
ATF6	-1.50	-1.67
MAP2K6	+1.79	+2.68

Of the genes regulated in the L1 kd tumors of both the initially L1CAM high (MeWo) and L1CAM low (MV3) cell lines (**Fig 6**), up-regulation of RFWD2 (RING finger and WD repeat domain 2, also COP1) in the L1 kd tumors should lead to an increase in p53/p21 pathway activity. This could be verified for the subcutaneous tumors of both cell lines using immunohistochemistry; however, there was a difference between the two cell lines: The L1CAM wildtype MeWo Luc tumors showed no p53 and low p21expression but the respective L1CAM knockdown tumors displayed clear p53 and p21 expression. In contrast to the MeWo Luc tumors, a pronounced p53 expression was found for the MV Luc tumors which did not change for in the L1 kd tumors. However, as for the MeWo tumors, p21 expression did increase in the MV3 L1 kd tumors (**S1 Fig**). According to the presumption that tumor cells with increased p53/p21 pathway activity should display a marked reduction in their metastatic potential, the remaining metastasis in the lungs of the L1 kd animals indeed showed no pronounced change in p53/p21 expression compared with their Luc counterparts with unchanged L1CAM expression (**S2 Fig**).

The only up-regulated gene of the 17 –MAP2K6 (dual specificity mitogen-activated protein kinase kinase 6) indicated an increase in p38 phosphorylation in the L1 kd tumors. The subcutaneous tumors for both cell lines showed an extreme intra-tumoral heterogeneity in p38 phosphorylation ranging from areas with extremely high phospho-p38 to areas with no detectably phospho-p38 which made a reasonable analysis by immunohistochemistry impossible. The L1 kd metastases from both cell lines, however, displayed an increase in phospho-p38 compared with their Luc counterparts (**S3 Fig**).

Summarized, L1CAM knockdown in the originally L1CAM high human melanoma cell line MeWo led to a reduced expression of EMT-related genes in the respective xenograft tumors (which also displayed reduced metastatic potential). An exception to this was the expression of the Notch ligand Jagged1 which showed an increase in expression. Genes with changed expression in the xenograft tumors after L1CAM knockdown for both melanoma cell lines indicate an increase in p53/p21 and also p38 activity which could be verified on protein level using immunohistochemistry. Both, reduced expression of EMT-network genes and increased p53/p21 and P38 activity probably contribute to the reduction of the metastatic potential of the human melanoma cells after L1CAM knockdown.

## Discussion

The performed *in vitro* experiments with the MeWo and MV3 Luc and L1CAM knockdown cells gave no clear indication how L1CAM influenced the metastatic behavior of the corresponding xenograft tumors *in vivo* (**Fig 1**) as both cell lines displayed different changes, e.g. in proliferation, after L1CAM knockdown. Melanoma cells are rather heterogenic in many aspects (in one and the same melanoma and also between two different ones)[22] which might in part explain why the *in vitro* changes in behavior differed between the two cell lines. We used an originally L1CAM high (MeWo) and an L1CAM low (MV3) cell line which might have been differently affected by L1CAM knockdown as their original dependence on L1CAM probably also differed. L1CAM knockdown led to a decrease in *in vitro* proliferation for MV3, but obviously even increased tumor growth *in vivo*. However, the *in vitro* proliferation assay is performed with cells in 2D culture with a standard cell culture medium. The conditions in this assay are thus quite different from the growth conditions in the animals and it appears not always to be very helpful for predicting the *in vivo* behavior of the cells. Considering the conflicting *in vitro* data we think that it is all the more remarkable that the influence of the L1CAM knockdown on the *in vivo* metastatic potential did not differ between the cell lines as the xenograft model of this study clearly demonstrated that L1CAM expression promotes melanoma metastasis. The unchanged CTC numbers in the xenograft indicate that the number of cells that left the primary tumor and entered the bloodstream (to become CTC) and were able to leave the vasculature to invade underlying tissue indeed did not differ between Luc and L1 kd cells. However, this number might not be directly correlated with the number of metastases as the L1CAM knockdown probably affects steps of the metastatic cascade following extravasation as the increase in p38 phosphorylation (**S3 Fig**) indicates that the melanoma cells in the L1 kd metastases are more stressed than the Luc controls, but L1CAM expression (albeit lower than in the luc controls) could be detected even in the metastases formed by the L1CAM knockdown cells (**Fig 3**) indicating that L1CAM is necessary for the outgrowth of metastases. Additionally, involvement of L1CAM in spreading on the abluminal surface of capillaries has been demonstrated in a xenograft model of brain metastasis with human breast and lung cancer cells [23].

Although melanocytes reside in the stratum basale of the epidermis they are not epithelial cells themselves but are derived from the neuronal crest. During malignant transformation these non-epthelial cells do not undergo a classic EMT (as observed in carcinoma), however, many molecules involved in EMT do also play important roles in the malignant transformation of melanocytes although these roles are sometimes different from classic EMT [24]. For example TWIST1 and ZEB1 increase the metastatic potential of melanoma cells whereas SNAI2 (SLUG) and ZEB2 decrease it [25]. According to our data from this study, L1CAM plays a functional role in melanoma metastasis and can influence the expression of “EMT” genes, however, this role appears to be in later stages of metastasis rather than in earlier stages as would be expected of a “classic” EMT molecule. High L1CAM expression in melanoma can obviously modulate the network of EMT gene expression in a pro-metastatic way with a strong influence on TGFβ signaling (**Fig 4**). Interestingly, L1CAM is able to directly or indirectly regulate TGFB1 expression and not only vice versa.

For L1CAM low melanoma, a partial compensation for the lack of L1CAM could be an overexpression of JAG1 (Jagged 1), modulating Notch signaling [26]. Upregulation of JAG1 is accompanied by increased Notch and CNTN1 expression indicating that the tumor cells themselves were, at least in part, target of the increased Jagged 1 signaling as there obviously were interactions between tumor cells via Jagged 1 / Notch. Downregulation of CCNC, HIF1A and KAT2B argue for a modulation of NOTCH signaling rather than for a simple increase. Interestingly, the initially L1CAM low MV3 tumors display a high expression of Jagged 1 from the start and further reduction of L1CAM does not lead to a further increase in Jagged 1. Probably, the expression of this Notch ligand cannot be increased beyond a certain point without negative effects for the melanoma cells. Jagged 1 was expressed in lung metastases in all four groups of animals in the xenograft experiment and Jagged 1 has been shown to be overexpressed in melanoma [27]. These findings make it plausible that Jagged 1 expression is promoting metastatic colonization and could thus be another interesting target for melanoma xenograft studies.

Of the genes regulated in the L1 kd tumors of both the initially L1CAM high and L1CAM low cell lines (**Fig 6**), RASAL2 (Ras GTPase-activating protein nGAP) and MALAT2 (long non-coding RNA Metastasis Associated Lung Adenocarcinoma Transcript 2) have been implicated in EMT networking, with the role of RASAL2 still under discussion [28–32]. Regulation of RFWD2 (RING finger and WD repeat domain 2, also COP1) and–the only up-regulated gene of the 17 –MAP2K6 (dual specificity mitogen-activated protein kinase kinase 6) indicate an increase in p53 [33] and p38 [34] activity and thus lower metastatic potential in the L1 kd tumors. Changes in the p53/p21 (**S1 and S2 Figs**) and p38 (**S3 Fig**) pathways could indeed be verified by immunhiostchemistry.

According to our results, L1CAM could be a promising target for combination therapy of human melanoma as a knockdown of L1CAM alone already led to a highly significant decrease in metastasis in the xenograft model of human melanoma described in this study even without changes in glycoprotein core-fucosylation by FUT8[12]. Interestingly, this decrease was observed for melanoma cells with originally high L1CAM expression as well as for melanoma cells with initially low L1CAM expression. Obviously, a considerable reduction in L1CAM causes a serious disruption of the highly complex intra- and/or intercellular signaling network determining the melanoma cells’ metastatic potential. The fact that the melanoma cells were not able to fully compensate the partial loss of L1CAM makes this CAM a highly interesting therapeutic target to prevent further metastatic spread in patients. It might be more feasible to interfere in L1CAM expression (e.g. by small molecule inhibitors) which would affect “only” L1CAM dependent cells than to interfere in glycoprotein core-fucosylation which would hit every cell in the human body. L1Cam could be an especially interesting target, as even patients with L1CAM low melanoma could profit from anti-L1CAM treatment.

## Supporting information

S1 Fig Human melanoma xenograft model: Immunohistochemical analysis reveals increased p53/p21 pathway activity in the subcutaneous tumors with L1CAM knockdown(L1 kd). Immunohistochemical staining for p53 or p21 expression (red) in subcutaneous (s.c.) tumors of human melanoma cells (MeWo and MV3) with unchanged L1CAM expression (Luc, respective upper panels) and L1CAM knockdown (L1 kd, respective lower panels). All scale bars: 100 μm. Stainings show ncreased p53 for MeWo L1 kd tumors only but increased p21 for MeWo and MV3 L1 kd tumors.
(TIF)

S2 Fig Human melanoma xenograft model: Immunohistochemical analysis shows no increase in p53/p21 in the remaining lung metastases for animals with L1CAM knockdown (L1 kd) tumors. Immunohistochemical staining for p53 (left panels) or p21 (right panels) expression (red) in lung metastases of human melanoma cells (MeWo and MV3) with unchanged L1CAM expression (Luc, respective upper panels) and L1CAM knockdown (L1 kd, respective lower panels). All scale bars: 50 μm. Stainings show no change in p53 and p21 expression for MeWo and MV3 L1 kd cells.
(TIF)

S3 Fig Human melanoma xenograft model: Immunohistochemical analysis shows an increase in phospho-p38 in the lung metastases for animals with L1CAM knockdown (L1 kd) tumors. Immunohistochemical staining for phospho-p38 (red) in lung metastases of human melanoma cells (MeWo and MV3) with unchanged L1CAM expression (Luc, respective upper panels) and L1CAM knockdown (respective lower panels). All scale bars: 50 μm.
(TIF)

S1 Table Comparison of the gene expression of subcutaneous MeWo tumors in scid mice with L1CAM knockdown (MeWo L1 kd) versus expression of MeWo Luc control (MeWo Luc) tumors. Included are only fold changes at least +/- 1.51; ANOVA and adjusted p-values <0.05.
(DOCX)

S2 Table Comparison of the gene expression of subcutaneous MV3 tumors in scid mice with L1CAM knockdown (MV3 L1 kd) versus expression of MV3 Luc control (MV3 Luc) tumors. Included are only fold changes at least +/- 1.51; ANOVA and adjusted p-values <0.05.
(DOCX)
