# Supplementary figures and images for: Knockdown of L1CAM significantly reduces metastasis in a xenograft model of human melanoma: L1CAM is a potential target for anti-melanoma therapy
Source: PLoS One. 2018 Feb 12;13(2):e0192525. doi: 10.1371/journal.pone.0192525 (PMC5809060; doi:10.1371/journal.pone.0192525)

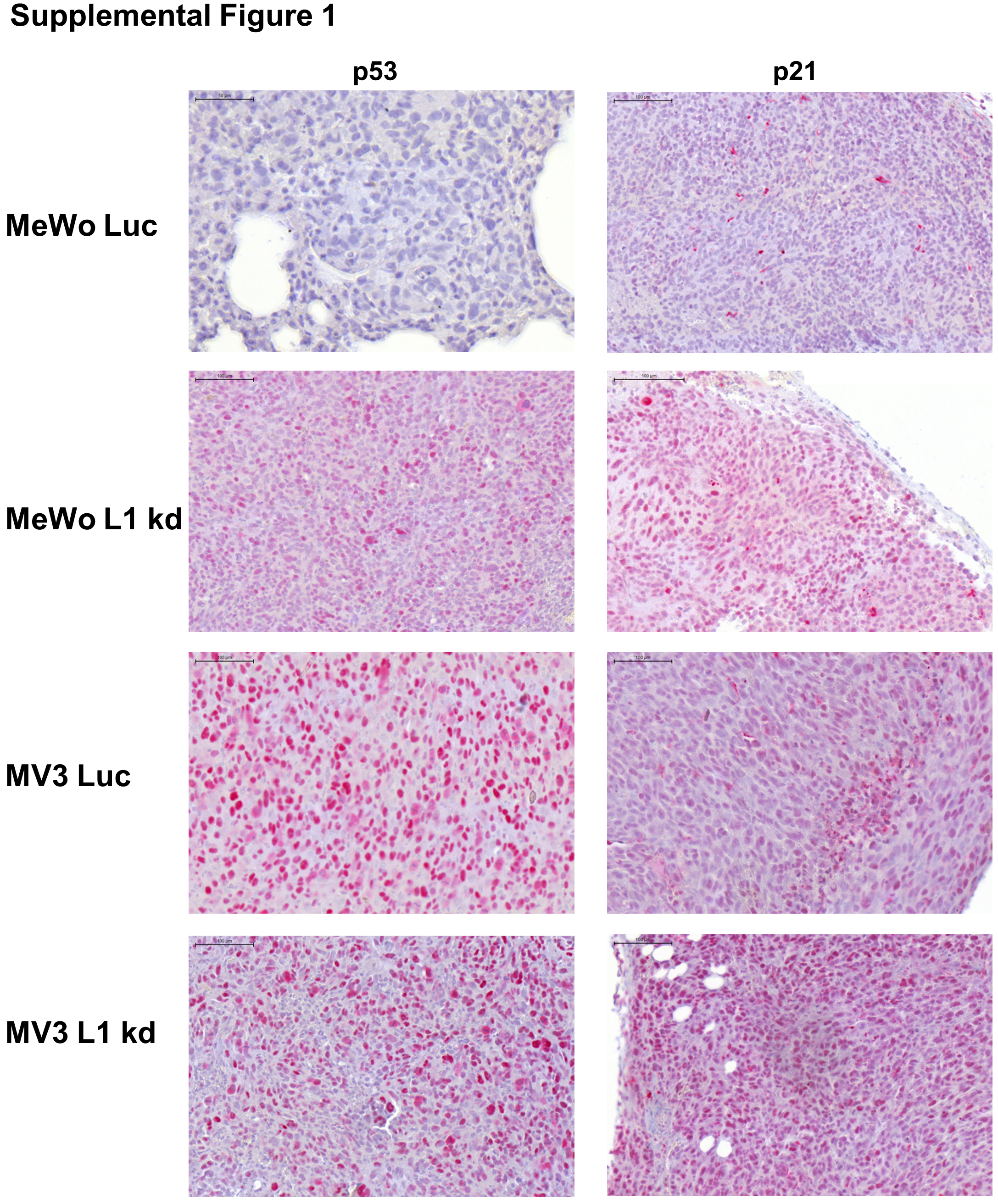

Supplement: S1 Fig — Immunohistochemical staining for p53 or p21 expression (red) in subcutaneous (s.c.) tumors of human melanoma cells (MeWo and MV3) with unchanged L1CAM expression (Luc, respective upper panels) and L1CAM knockdown (L1 kd, respective lower panels). All scale bars: 100 μm. Stainings show ncreased p53 for MeWo L1 kd tumors only but increased p21 for MeWo and MV3 L1 kd tumors. (TIF) [file pone.0192525.s001.tif]

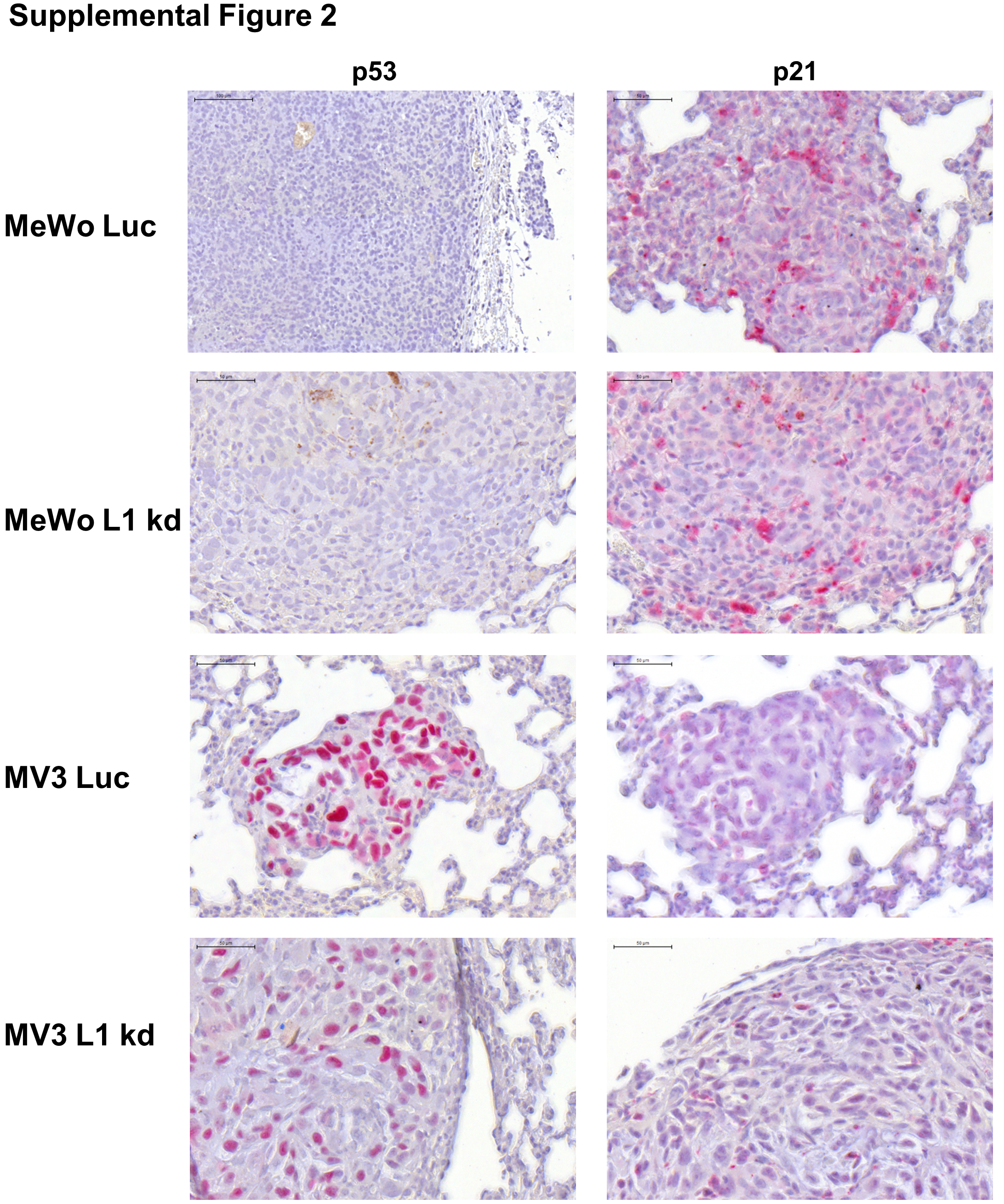

Supplement: S2 Fig — Immunohistochemical staining for p53 (left panels) or p21 (right panels) expression (red) in lung metastases of human melanoma cells (MeWo and MV3) with unchanged L1CAM expression (Luc, respective upper panels) and L1CAM knockdown (L1 kd, respective lower panels). All scale bars: 50 μm. Stainings show no change in p53 and p21 expression for MeWo and MV3 L1 kd cells. (TIF) [file pone.0192525.s002.tif]

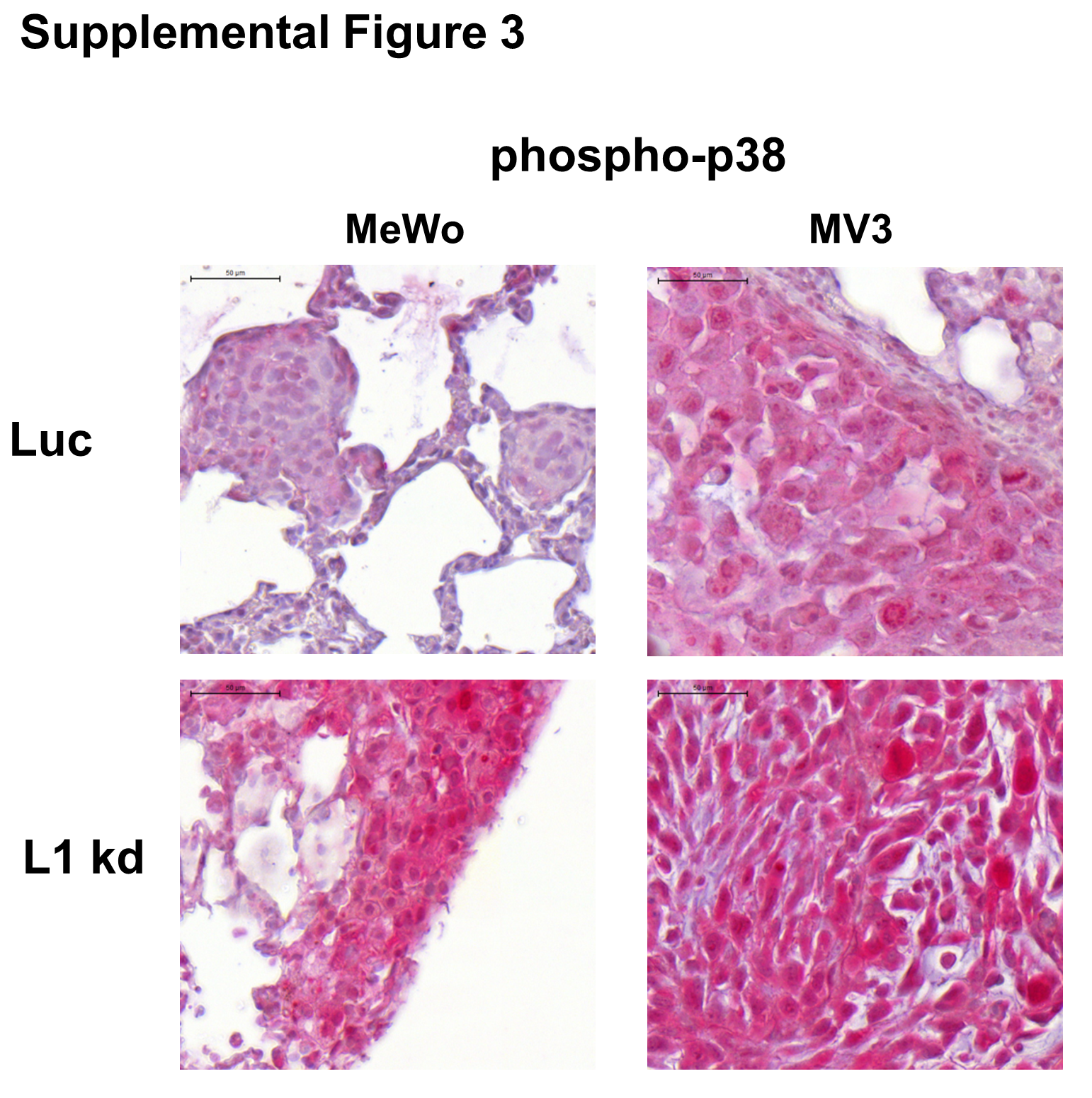

Supplement: S3 Fig — Immunohistochemical staining for phospho-p38 (red) in lung metastases of human melanoma cells (MeWo and MV3) with unchanged L1CAM expression (Luc, respective upper panels) and L1CAM knockdown (respective lower panels). All scale bars: 50 μm. (TIF) [file pone.0192525.s003.tif]
