# Supplementary material for: Knockdown of L1CAM significantly reduces metastasis in a xenograft model of human melanoma: L1CAM is a potential target for anti-melanoma therapy
Source: PLoS One. 2018 Feb 12;13(2):e0192525. doi: 10.1371/journal.pone.0192525 (PMC5809060; doi:10.1371/journal.pone.0192525)
Supplement: S1 Table — Included are only fold changes at least +/- 1.51; ANOVA and adjusted p-values <0.05. (DOCX) [file pone.0192525.s004.docx]

**Supplemental Table 1:** **Comparison of the gene expression of subcutaneous MeWo tumors in scid mice with L1CAM knockdown (MeWo L1 kd) versus expression of MeWo Luc control (MeWo Luc) tumors. Included are only fold changes at least +/- 1.51; ANOVA and adjusted p-values <0.05.**

| **gene symbol** | **fold change** |
| --- | --- |
| PLCH1-AS1 | 27.83 |
| DCT | 10.92 |
| MUC12 | 10.91 |
| USP17L23 | 10.42 |
| SLC38A1 | 8.71 |
| USP17L6P | 8.38 |
| SLC45A2 | 8.36 |
| RP1-81D8.3 | 8.17 |
| XYLT1 | 7.93 |
| EPHA3 | 7.71 |
| USP17L29; USP17L6P | 7.66 |
| USP17; USP17L25; USP17L24; USP17L5; USP17L29; USP17L26; USP17L27; USP17L28; USP17L30 | 7.39 |
| USP17; USP17L25; USP17L24; USP17L29; USP17L26; USP17L27; USP17L28; USP17L30 | 7.39 |
| USP17L25; USP17L24; USP17L26; USP17L29; USP17L27; USP17L28; USP17L30 | 7.39 |
| USP17L25; USP17L24; USP17L26; USP17L27; USP17L28; USP17L29; USP17L30 | 7.39 |
| RP11-181K12.2 | 7.31 |
| MUC4 | 7.26 |
| SNORD115-5; SNORD115-9; SNORD115-10; SNORD115-12 | 5.94 |
| SNORD115-11; SNORD115-29; SNORD115-43; SNORD115-36 | 5.77 |
| SNORD115-11; SNORD115-29; SNORD115-36; SNORD115-43 | 5.77 |
| USP17; USP17L25; USP17L24; USP17L29; USP17L5; USP17L30 | 5.7 |
| USP17L29; USP17L18; USP17L20 | 5.5 |
| USP17L21 | 5.47 |
| MIR3689A; MIR3689E | 5.47 |
| USP17; USP17L25; USP17L24; USP17L26; USP17L27; USP17L28; USP17L29; USP17L30 | 5.46 |
| SNORD115-22 | 5.28 |
| MIR3689C | 5.24 |
| REXO1L1; REXO1L2P; LOC100288562; LOC101929601; LOC101929627 | 5.09 |
| USP17; USP17L11; USP17L20; USP17L22; USP17L17 | 5.08 |
| USP17L12 | 5.03 |
| USP17L10; USP17L13 | 5.03 |
| SNORD115-15; SNORD115-20; SNORD115-21; SNORD115-34 | 4.72 |
| USP17L19 | 4.49 |
| ADRBK2 | 4.4 |
| RP11-34P13.9 | 4.39 |
| PTPRJ; LOC100287223 | 4.36 |
| AC009495.4 | 4.28 |
| RNU1-143P | 4.19 |
| HMCN1 | 4.16 |
| HIPK2 | 4.07 |
| REXO1L1; REXO1L2P; LOC100288562; LOC101929601; LOC101929627; REXO1L1P | 3.96 |
| MIR3689B | 3.91 |
| MIR297 | 3.8 |
| USP17L15 | 3.71 |
| XXbac-B33L19.6 | 3.69 |
| AC073218.3 | 3.69 |
| SNORD115-21 | 3.68 |
| REXO1L2P | 3.68 |
| ITGA9 | 3.61 |
| MIR548I2 | 3.58 |
| TOX3 | 3.55 |
| RP11-1018N14.3 | 3.48 |
| MIR548I3 | 3.45 |
| SEMA6D | 3.4 |
| MUC3A | 3.39 |
| SNORD115-10 | 3.38 |
| MIR548I1 | 3.36 |
| CST1 | 3.36 |
| AC104389.16 | 3.35 |
| TGFBR3 | 3.29 |
| SNORD115-10; SNORD115-42 | 3.27 |
| SH3BGRL2 | 3.25 |
| DNM1P47; DNM1P46 | 3.24 |
| SESN3 | 3.23 |
| PLTP | 3.22 |
| SNORD62A; SNORD62B; RP11-334J6.4 | 3.21 |
| SNORD62A; SNORD62B | 3.21 |
| SNORD115-25 | 3.19 |
| REXO1L1; REXO1L2P; REXO1L1P; LOC100288562 | 3.18 |
| SLC7A2 | 3.17 |
| RP4-669L17.2 | 3.17 |
| SNORD115-6 | 3.16 |
| MIR548I4 | 3.07 |
| MIR3689F | 3 |
| GNAO1 | 3 |
| SNORD115-16 | 2.99 |
| SNORD115-40 | 2.98 |
| CTD-2281E23.3 | 2.96 |
| SNORD115-3 | 2.94 |
| MIR548T | 2.94 |
| MIR548A3 | 2.91 |
| CEBPZOS | 2.88 |
| MIR548H2 | 2.87 |
| MIR548AA2 | 2.87 |
| LOC101928495; RP11-13E5.2 | 2.87 |
| ITM2A | 2.87 |
| MIR548O2 | 2.86 |
| RP11-380P13.2 | 2.83 |
| MIR548X | 2.83 |
| RP11-598D14.1 | 2.82 |
| POM121L9P | 2.81 |
| MIR548AJ2 | 2.81 |
| BPTF | 2.81 |
| USP17L1P | 2.8 |
| MIR548H3 | 2.79 |
| RP11-267L5.1 | 2.78 |
| RNA5SP315 | 2.78 |
| RNA5SP313 | 2.78 |
| RNA5SP312 | 2.78 |
| MIR4454 | 2.78 |
| CTSK | 2.78 |
| SNORD115-15 | 2.76 |
| PTGFRN | 2.75 |
| AC009495.3 | 2.74 |
| RP11-69I8.2 | 2.73 |
| FAR2 | 2.72 |
| RP1-266L20.4 | 2.71 |
| MIR548F1 | 2.71 |
| RP11-1144P22.1 | 2.7 |
| RP11-206L10.4 | 2.69 |
| MAP2K6 | 2.68 |
| SNORD115-41 | 2.67 |
| NEO1 | 2.67 |
| MIR548H4 | 2.63 |
| THBS2 | 2.62 |
| PTPRJ | 2.62 |
| ERVH-6 | 2.62 |
| ABCA9-AS1 | 2.61 |
| LINC00500 | 2.6 |
| MIR548AI | 2.59 |
| SNORD115-44 | 2.57 |
| RP11-353N4.4 | 2.56 |
| MIR548F3 | 2.56 |
| LTB | 2.56 |
| MIR3974 | 2.55 |
| PTPRS | 2.54 |
| PER3 | 2.54 |
| MIR548W | 2.54 |
| MIR548U | 2.54 |
| USP17L2; USP17L8; USP17L7; USP17L1P; USP17L4; USP17L3 | 2.52 |
| SNORD115-39 | 2.52 |
| MUC17 | 2.52 |
| MIR548A2 | 2.52 |
| ADAM23 | 2.52 |
| ABCC2 | 2.52 |
| PHLPP1 | 2.51 |
| MIR548G | 2.51 |
| RNU7-53P | 2.5 |
| REXO1L1; REXO1L2P | 2.5 |
| IGKV2D-28 | 2.48 |
| DUX4 | 2.48 |
| SNORD115-17; SNORD115-18; SNORD115-19 | 2.47 |
| MIR548AN | 2.47 |
| CTD-2501M5.1 | 2.47 |
| RP11-154F14.2 | 2.44 |
| DCAF7 | 2.44 |
| SNORD115-31 | 2.43 |
| SNORD115-1 | 2.43 |
| RNU6-429P | 2.4 |
| LOC101929450; AC145110.1 | 2.4 |
| CASC9 | 2.4 |
| AC011718.3 | 2.4 |
| RP11-763B22.6 | 2.36 |
| RP11-400N13.2 | 2.36 |
| IGKV1D-27 | 2.36 |
| TNC | 2.35 |
| SNORD115-14 | 2.35 |
| LINC00965 | 2.35 |
| JAG1 | 2.35 |
| AC107079.1 | 2.35 |
| SEMA5A | 2.34 |
| SNORA74A | 2.33 |
| UBE2O | 2.32 |
| RP11-431N15.2 | 2.28 |
| RNA5SP316 | 2.28 |
| RNA5SP310 | 2.28 |
| CREB5 | 2.28 |
| RP11-423O2.7 | 2.27 |
| RP11-217C7.1 | 2.27 |
| MIR1299 | 2.27 |
| DUSP9 | 2.27 |
| CST4 | 2.27 |
| NLGN4Y | 2.26 |
| MIR548Q | 2.26 |
| LINC01183; CTC-228N24.1 | 2.26 |
| RNA5SP317 | 2.25 |
| RNA5SP314 | 2.25 |
| RNA5SP311 | 2.25 |
| SEMA6A | 2.24 |
| CSF2RA | 2.24 |
| TLR8-AS1 | 2.22 |
| RP11-434D9.2 | 2.22 |
| LOC146481 | 2.22 |
| AC009229.6 | 2.22 |
| BX004987.7 | 2.21 |
| SNX33 | 2.2 |
| GAS7 | 2.2 |
| ADCY2 | 2.2 |
| RP11-159F24.6 | 2.19 |
| PPM1K | 2.19 |
| SUPT6H | 2.18 |
| BX571672.4 | 2.18 |
| ANK3 | 2.18 |
| ZZEF1 | 2.17 |
| OR4F5 | 2.17 |
| TYRP1 | 2.16 |
| RP11-51P8.1 | 2.16 |
| HLA-C | 2.16 |
| OR2A7 | 2.15 |
| CALB1 | 2.15 |
| RNU7-24P | 2.14 |
| IGKC | 2.14 |
| LOC339539 | 2.13 |
| AC104088.1 | 2.13 |
| SNORD1A; SNORD1C; SNHG16 | 2.12 |
| OR7E5P | 2.12 |
| LOC100132859; LOC100289375; LOC100509638; FAM27D1 | 2.12 |
| LINC01067; RP11-259F4.1 | 2.12 |
| GATAD2B | 2.12 |
| RP11-403I13.9 | 2.11 |
| LINC00452 | 2.11 |
| KRTAP5-11 | 2.11 |
| GNAL | 2.11 |
| FERMT1 | 2.11 |
| MIR548AC | 2.1 |
| LOC440173 | 2.1 |
| TRIM63 | 2.09 |
| RP4-660H19.1 | 2.09 |
| RNU7-156P | 2.09 |
| MIR548D2 | 2.09 |
| MIR548AL | 2.09 |
| GABRG3 | 2.09 |
| EGR1 | 2.09 |
| AC092669.3 | 2.09 |
| SNORD115-4 | 2.08 |
| RNU2-55P | 2.08 |
| OR2M3 | 2.08 |
| MIR548M | 2.08 |
| MERTK | 2.08 |
| CEBPZOS; AC007390.5 | 2.08 |
| BX004987.6 | 2.08 |
| BAAT | 2.08 |
| AC008278.3 | 2.08 |
| RNU6-1304P | 2.07 |
| OTUD7B | 2.07 |
| IGHD2-15 | 2.07 |
| MIR548AD | 2.06 |
| LOC101930011; LOC101930433 | 2.06 |
| LCE2A | 2.06 |
| AC005152.3 | 2.06 |
| SMARCD2 | 2.05 |
| SLC9A1 | 2.05 |
| RP4-760C5.3 | 2.05 |
| OR1D4 | 2.05 |
| RP11-318G21.4 | 2.04 |
| RP11-158J3.2 | 2.04 |
| RNU6-558P | 2.04 |
| PRKCA | 2.04 |
| OR7E91P | 2.04 |
| OR2A4 | 2.04 |
| RP11-83M16.6 | 2.03 |
| RNU7-106P | 2.03 |
| RNU6-348P | 2.03 |
| LOC100507330 | 2.03 |
| CTD-2013M15.1 | 2.03 |
| CRIPAK | 2.03 |
| KIAA0196-AS1 | 2.02 |
| KB-1592A4.14 | 2.02 |
| AC005150.1 | 2.02 |
| SV2A | 2.01 |
| RNU6-426P | 2.01 |
| RNU6-1248P | 2.01 |
| NUP214 | 2.01 |
| HYDIN2 | 2.01 |
| AC018804.3 | 2.01 |
| RNU7-16P | 2 |
| PRDX2 | 2 |
| ST8SIA1 | 1.99 |
| RP11-353N4.5 | 1.99 |
| PRELP | 1.99 |
| PODXL2 | 1.99 |
| OR6K2 | 1.99 |
| OR2A9P; OR2A20P | 1.99 |
| RNU7-75P | 1.98 |
| RNU6-24P | 1.98 |
| MIR548Z | 1.98 |
| MIR548D2; MIR548AA2 | 1.98 |
| GALNT3 | 1.98 |
| CECR2 | 1.98 |
| APOD | 1.98 |
| AC144450.1 | 1.98 |
| RP11-393K12.2 | 1.97 |
| RAB6B | 1.97 |
| MST1L | 1.97 |
| ZBTB40 | 1.96 |
| RP11-1217F2.15 | 1.96 |
| LINC00350 | 1.96 |
| GAGE2B; GAGE12B; GAGE2C | 1.96 |
| CEP170P1 | 1.96 |
| ERV9-1 | 1.95 |
| ARL17A | 1.95 |
| ADCY1 | 1.95 |
| SLC38A8 | 1.94 |
| RP11-561O23.7 | 1.94 |
| FAM90A12P | 1.94 |
| ASH1L; MIR555 | 1.94 |
| AC005276.1 | 1.94 |
| C1orf233 | 1.93 |
| ZNF521 | 1.92 |
| PRKAA2 | 1.92 |
| MIR9-1 | 1.92 |
| LOC101928739; RP11-360P21.2 | 1.92 |
| IGKV2-28 | 1.92 |
| GSTM4 | 1.92 |
| EGR3 | 1.92 |
| AL583842.2 | 1.92 |
| AC002539.1 | 1.92 |
| UBR4 | 1.91 |
| RNU7-62P | 1.91 |
| RNU4-72P | 1.91 |
| NAV3 | 1.91 |
| AL583842.1 | 1.91 |
| AC069277.2 | 1.91 |
| RNU6-80P | 1.9 |
| RNU6-232P | 1.9 |
| PDLIM1 | 1.9 |
| OR9A1P | 1.9 |
| MIR548H1 | 1.9 |
| MIR4441 | 1.9 |
| LINC-ROR | 1.9 |
| KMT2A; MLL | 1.9 |
| TULP4 | 1.89 |
| PLEKHG3 | 1.89 |
| LINC01314 | 1.89 |
| LCE2B | 1.89 |
| FTSJ3 | 1.89 |
| TRPC7-AS2 | 1.88 |
| TNRC6C | 1.88 |
| IGSF11 | 1.88 |
| EP300; MIR1281 | 1.88 |
| SNORA5A | 1.87 |
| RP11-447M12.2 | 1.87 |
| RNU7-20P | 1.87 |
| MGC32805; CTC-210G5.1 | 1.87 |
| MCC | 1.87 |
| LOC729461 | 1.87 |
| IGHV3OR16-10 | 1.87 |
| TANC2 | 1.86 |
| RP5-1185H19.2 | 1.86 |
| RP11-540H22.2 | 1.86 |
| RNU6-21P | 1.86 |
| RNU6-1075P | 1.86 |
| PRPF8 | 1.86 |
| PRKDC | 1.86 |
| POLR1A | 1.86 |
| MIR4466 | 1.86 |
| HUWE1 | 1.86 |
| FAM90A24P | 1.86 |
| CYSLTR2 | 1.86 |
| RP11-397D12.4 | 1.85 |
| RNU6-1084P | 1.85 |
| RNU6-31P | 1.84 |
| MIR548D1 | 1.84 |
| GAGE2D; GAGE2E; GAGE2A; GAGE2C; GAGE8 | 1.84 |
| FAM138B; FAM138D | 1.84 |
| DST | 1.84 |
| CTB-17P3.4 | 1.84 |
| RNU6-905P | 1.83 |
| RNU6-550P | 1.83 |
| MIR4524A | 1.83 |
| LOC100652750; LOC729461; AC008079.9 | 1.83 |
| GRAMD4 | 1.83 |
| BTBD2 | 1.83 |
| SPTAN1 | 1.82 |
| RP1-182D15.2 | 1.82 |
| RP11-139I14.2 | 1.82 |
| RNU6-744P | 1.82 |
| RNU6-562P | 1.82 |
| RNU6-216P | 1.82 |
| RNU6-1145P | 1.82 |
| MYO5A | 1.82 |
| MIR539 | 1.82 |
| DUX4L3; DUX4; DUX4L | 1.82 |
| AHCYL2 | 1.82 |
| AC010145.4 | 1.82 |
| RP11-527D7.1 | 1.81 |
| RP11-432J24.2 | 1.81 |
| MIR1255B2 | 1.81 |
| GAGE12J; GAGE4; GAGE5; GAGE6; GAGE12G; GAGE2D; GAGE12C; GAGE12E; GAGE12H; GAGE12F; GAGE12D | 1.81 |
| ARRB1 | 1.81 |
| AC097532.2 | 1.81 |
| AC019100.3 | 1.81 |
| ZSWIM6 | 1.8 |
| SRRM2 | 1.8 |
| SEPT4 | 1.8 |
| RP11-64C1.1 | 1.8 |
| RNU7-48P | 1.8 |
| PRKD3 | 1.8 |
| LOC101929470; RP11-94H18.1 | 1.8 |
| GAGE12F; GAGE2B; GAGE2E; GAGE2A; GAGE2C; GAGE4; GAGE5; GAGE12I; GAGE8; GAGE7; GAGE12G; GAGE12B | 1.8 |
| SLC9A9-AS2 | 1.79 |
| RNU7-165P | 1.79 |
| PTPRM | 1.79 |
| INTS2 | 1.79 |
| GAREM | 1.79 |
| CTA-929C8.5 | 1.79 |
| CLSTN1 | 1.79 |
| ARID1A | 1.79 |
| USP17L4 | 1.78 |
| UCA1 | 1.78 |
| RP11-466P24.6 | 1.78 |
| RNU6-180P | 1.78 |
| RNU6-1287P | 1.78 |
| RNU6-1209P | 1.78 |
| RNU6-1183P | 1.78 |
| PRR21 | 1.78 |
| MIR3689D2 | 1.78 |
| LOC650293 | 1.78 |
| KCNJ13 | 1.78 |
| ERVH-1 | 1.78 |
| DEFB108P2 | 1.78 |
| CDK12 | 1.78 |
| RP11-144G6.10 | 1.77 |
| RNU6-1152P | 1.77 |
| KMT2D | 1.77 |
| DUSP5P1 | 1.77 |
| AP002856.7 | 1.77 |
| AP001464.4 | 1.77 |
| RP11-38J22.1 | 1.76 |
| RNU6-911P | 1.76 |
| RNU6-580P | 1.76 |
| NAV1 | 1.76 |
| IGF1R | 1.76 |
| GIMAP4 | 1.76 |
| GAGE13; GAGE2E; GAGE8; GAGE2A | 1.76 |
| CNOT1 | 1.76 |
| VEPH1 | 1.75 |
| TRRAP | 1.75 |
| SPRR2A | 1.75 |
| SPEN | 1.75 |
| SNORA57 | 1.75 |
| SCPEP1 | 1.75 |
| RP11-96C23.10 | 1.75 |
| RNU7-182P | 1.75 |
| RNU6-622P | 1.75 |
| RNU6-1256P | 1.75 |
| KMT2A | 1.75 |
| IGHV3OR16-9 | 1.75 |
| GAGE2D; GAGE2C; GAGE2B; GAGE2E; GAGE2A | 1.75 |
| AC009878.2 | 1.75 |
| RP11-219C24.10 | 1.74 |
| RP11-143M1.3 | 1.74 |
| RNU7-179P | 1.74 |
| RNU7-155P | 1.74 |
| RNU6-672P | 1.74 |
| RNU6-59P | 1.74 |
| RNU6-224P | 1.74 |
| RNU6-1241P | 1.74 |
| MIR610 | 1.74 |
| MGC72080 | 1.74 |
| MGA | 1.74 |
| LINC00444 | 1.74 |
| IGKV1-9 | 1.74 |
| ASPA | 1.74 |
| RP11-524K22.1 | 1.73 |
| RP11-347D21.1 | 1.73 |
| RNU6-235P | 1.73 |
| RNU6-227P | 1.73 |
| RNU6-1123P | 1.73 |
| PDCD11 | 1.73 |
| MKL2 | 1.73 |
| ESRG | 1.73 |
| DDX11L9; DDX11L1; DDX11L5; DDX11L12; DDX11L11 | 1.73 |
| RP5-827L5.1 | 1.72 |
| RP4-781K5.6 | 1.72 |
| RP11-331F9.3 | 1.72 |
| RNU6-1340P | 1.72 |
| OR4F2P | 1.72 |
| LOC101927881 | 1.72 |
| LINC00415 | 1.72 |
| DIP2B | 1.72 |
| CTC-458G6.4 | 1.72 |
| CREBBP | 1.72 |
| TP53TG3D; TP53TG3; TP53TG3C; TP53TG3B | 1.71 |
| SRGAP2-AS1 | 1.71 |
| RP13-221M14.2 | 1.71 |
| RP11-946L20.2 | 1.71 |
| RNU7-57P | 1.71 |
| RNU6-742P | 1.71 |
| RNU6-44P | 1.71 |
| RNU6-242P | 1.71 |
| RNU6-166P | 1.71 |
| RNU6-1158P | 1.71 |
| RNU6-1090P | 1.71 |
| NAPEPLD | 1.71 |
| MOB3B | 1.71 |
| MLLT10P1 | 1.71 |
| LINC01377; CTD-2029E14.1 | 1.71 |
| KB-1183D5.14 | 1.71 |
| GAGE2A; GAGE2C | 1.71 |
| DUX4L2; DUX4L4; DUX2; DUX4; DUX4L1; DUX4L8; DUX4L7; DUX4L6; DUX4L5; DUX4L3; LOC100288289 | 1.71 |
| RNU6-868P | 1.7 |
| RNU6-38P | 1.7 |
| RNU6-318P | 1.7 |
| RNU6-266P | 1.7 |
| NETO2 | 1.7 |
| MAP3K3 | 1.7 |
| LPA | 1.7 |
| LINC00525 | 1.7 |
| LINC00368; RP11-551M15.1 | 1.7 |
| EIF4A3 | 1.7 |
| CHD7 | 1.7 |
| CAPN5 | 1.7 |
| AC012506.4 | 1.7 |
| SMG1 | 1.69 |
| RP11-347L18.1 | 1.69 |
| RP11-144G6.4 | 1.69 |
| RP11-1145L24.1 | 1.69 |
| RNU6-948P | 1.69 |
| RNU6-647P | 1.69 |
| RNU6-373P | 1.69 |
| RNU6-199P | 1.69 |
| RNU6-1316P | 1.69 |
| RNU6-10P | 1.69 |
| RNU6-1047P | 1.69 |
| LOC101927888; RP3-527G5.1 | 1.69 |
| FAM138C | 1.69 |
| DGCR5 | 1.69 |
| AC012593.1 | 1.69 |
| USP36 | 1.68 |
| TP53TG3B; TP53TG3C; TP53TG3; TP53TG3D | 1.68 |
| RP5-1132H15.1 | 1.68 |
| RP11-545D19.1 | 1.68 |
| RNU6-964P | 1.68 |
| RNU6-936P | 1.68 |
| RNU6-698P | 1.68 |
| RNU6-1312P | 1.68 |
| RNU6-1042P | 1.68 |
| RNA5SP468 | 1.68 |
| RNA5SP153 | 1.68 |
| RFX7 | 1.68 |
| RASIP1 | 1.68 |
| PARP1 | 1.68 |
| MED13 | 1.68 |
| LOC101928381 | 1.68 |
| LOC101927123; RP11-88I21.2 | 1.68 |
| ENPP7P8 | 1.68 |
| ATP11A | 1.68 |
| AQP7P3 | 1.68 |
| AC010729.2 | 1.68 |
| TARS2; MIR6878 | 1.67 |
| SRGAP2-AS1; RP11-343N15.1 | 1.67 |
| RP11-534L6.2 | 1.67 |
| RNU6-921P | 1.67 |
| RNU6-1190P | 1.67 |
| RNU6-1011P | 1.67 |
| RNA5SP320 | 1.67 |
| RMDN2-AS1 | 1.67 |
| PRR20B; PRR20C; PRR20D; PRR20E; PRR20A | 1.67 |
| OR9A4 | 1.67 |
| OR2L2 | 1.67 |
| MGAT5 | 1.67 |
| MAP3K1 | 1.67 |
| LOC101927827; RP11-111F5.8 | 1.67 |
| IGKV2-24 | 1.67 |
| HEATR1 | 1.67 |
| GDPD5 | 1.67 |
| AL022344.4 | 1.67 |
| AC144450.2 | 1.67 |
| AC092660.1 | 1.67 |
| TMTC1 | 1.66 |
| SNRNP200; LOC101929240 | 1.66 |
| RP5-997D16.2 | 1.66 |
| RP11-66D17.5 | 1.66 |
| RP11-44H4.1 | 1.66 |
| RNU6-737P | 1.66 |
| RNU6-734P | 1.66 |
| RNU6-463P | 1.66 |
| RNU6-1122P | 1.66 |
| RNU5E-1; RNU5D-1 | 1.66 |
| MIR190B | 1.66 |
| MIR1285-2 | 1.66 |
| JARID2 | 1.66 |
| IL6R | 1.66 |
| TNF | 1.65 |
| RP11-445P17.3 | 1.65 |
| RNU7-55P | 1.65 |
| RNU6-220P; RP5-1118A7.3 | 1.65 |
| RNU6-1188P | 1.65 |
| RNU6-1020P | 1.65 |
| RNU5F-1 | 1.65 |
| PTPRF | 1.65 |
| POM121L8P; POM121L4P; LOC727983; AC008132.13 | 1.65 |
| OR2M7 | 1.65 |
| LOC101929515; RP11-552E20.4 | 1.65 |
| LOC100506393 | 1.65 |
| LOC100505811; CTD-3179P9.2 | 1.65 |
| GAGE1 | 1.65 |
| DUX4L2; DUX4L6; DUX2; DUX4L1; DUX4L8; DUX4L7; DUX4L5; DUX4L3 | 1.65 |
| CTD-3023L14.3 | 1.65 |
| CEP170 | 1.65 |
| AC092415.1 | 1.65 |
| UBR5 | 1.64 |
| TXNIP; LOC101060503 | 1.64 |
| SEC14L1; SCARNA16; SNHG20; MIR6516; LINC00338 | 1.64 |
| RP4-704D21.2 | 1.64 |
| RP11-152K4.2 | 1.64 |
| RP11-108M9.2 | 1.64 |
| RNU6-40P | 1.64 |
| RNU6-211P | 1.64 |
| POU6F2-AS2 | 1.64 |
| OR7E37P | 1.64 |
| OR4C13 | 1.64 |
| OR1L3 | 1.64 |
| MYC | 1.64 |
| MIR656 | 1.64 |
| MANEAL | 1.64 |
| LOC100507194; RP1-40E16.9 | 1.64 |
| KIAA0430 | 1.64 |
| DDX11L9 | 1.64 |
| CXorf51B | 1.64 |
| CHD4 | 1.64 |
| SCGB3A2 | 1.63 |
| RNU7-141P | 1.63 |
| RNU6-961P | 1.63 |
| RNU6-794P | 1.63 |
| RNU6-416P | 1.63 |
| RNU6-19P | 1.63 |
| RNU6-1060P | 1.63 |
| RNU6-1024P | 1.63 |
| PDCD4-AS1 | 1.63 |
| OR4P4 | 1.63 |
| OR2T8 | 1.63 |
| MIR548F4 | 1.63 |
| LOC102467226; CTC-546K23.1 | 1.63 |
| LOC100507556 | 1.63 |
| LINC01189; LOC101927827; RP11-475I24.8 | 1.63 |
| LINC00458 | 1.63 |
| LCE2C | 1.63 |
| GAGE12F; GAGE12C; GAGE12D; GAGE6; GAGE12I; GAGE4; GAGE5; GAGE7; GAGE12G; GAGE12B | 1.63 |
| CTC-286N12.1 | 1.63 |
| CNDP2 | 1.63 |
| ACTR3BP6 | 1.63 |
| AC118653.2 | 1.63 |
| AC012668.3 | 1.63 |
| SNORD115-38 | 1.62 |
| SNORD115-30 | 1.62 |
| SNORD115-24 | 1.62 |
| SNORD114-7 | 1.62 |
| SF3B3 | 1.62 |
| RPRD2 | 1.62 |
| RP11-85L21.6 | 1.62 |
| RNU7-73P | 1.62 |
| RNU6-840P | 1.62 |
| RNU6-748P | 1.62 |
| RNU6-43P | 1.62 |
| RNU6-326P | 1.62 |
| RNU6-296P | 1.62 |
| RNU6-271P | 1.62 |
| POLR2A | 1.62 |
| NR4A3 | 1.62 |
| MIR509-1; MIR509-2; MIR509-3 | 1.62 |
| KDM2A | 1.62 |
| FAM138A; FAM138F; FAM138C | 1.62 |
| ANK2 | 1.62 |
| TRAM2 | 1.61 |
| SPRR1A | 1.61 |
| SGK2 | 1.61 |
| RP11-5K23.5 | 1.61 |
| RP11-347D21.2 | 1.61 |
| RNU6-838P | 1.61 |
| RNU6-777P | 1.61 |
| RNU6-727P | 1.61 |
| RNU6-589P | 1.61 |
| RNU6-49P | 1.61 |
| RNU6-32P | 1.61 |
| RNU6-13P | 1.61 |
| RNU6-12P | 1.61 |
| POGZ | 1.61 |
| MIR4320 | 1.61 |
| MDH2 | 1.61 |
| LSAMP; RP11-384F7.2 | 1.61 |
| IGLJ6 | 1.61 |
| IGHV3-66 | 1.61 |
| FAM230B | 1.61 |
| EP400 | 1.61 |
| DYNC1H1 | 1.61 |
| DUX4L6; DUX4L5; DUX4L3; DUX4L4; DUX4; DUX2; DUX4L7; DUX4L2; LOC100288355; LOC100288398; LOC100288433; LOC100288494; LOC100288593; LOC100288657; DUX4L1; DUX4L8; LOC652301; LOC100291626; LOC100288627 | 1.61 |
| DMXL2 | 1.61 |
| BACH1-AS1 | 1.61 |
| AJ239322.1 | 1.61 |
| TEX26-AS1 | 1.6 |
| RP1-149A16.12 | 1.6 |
| RP11-164C1.2 | 1.6 |
| RNU6-758P | 1.6 |
| RNU6-41P | 1.6 |
| RNU6-188P | 1.6 |
| MIR3148 | 1.6 |
| LOC728073 | 1.6 |
| LOC100506533 | 1.6 |
| HEG1 | 1.6 |
| FOXP2 | 1.6 |
| DDX11L1; DDX11L5; DDX11L12; DDX11L9 | 1.6 |
| ATP9A | 1.6 |
| AC109826.1 | 1.6 |
| AC098973.2 | 1.6 |
| AC009276.4 | 1.6 |
| RP4-760C5.5 | 1.59 |
| RP3-388N13.3 | 1.59 |
| RP11-775B15.2 | 1.59 |
| RP11-758M4.4 | 1.59 |
| RNU7-92P | 1.59 |
| RNU6-97P | 1.59 |
| RNU6-677P | 1.59 |
| RNU6-574P | 1.59 |
| RNU6-47P | 1.59 |
| RNU6-23P | 1.59 |
| RNU6-1327P | 1.59 |
| RNU6-1181P | 1.59 |
| RNU6-1013P | 1.59 |
| PPM1K; RNU6-112P | 1.59 |
| OR2AG1 | 1.59 |
| MIR603 | 1.59 |
| LOC100508046; LOC101929572; POTEH-AS1 | 1.59 |
| LINC01419; RP11-51M18.1 | 1.59 |
| IGLJ5 | 1.59 |
| IGKV2D-26 | 1.59 |
| FASN | 1.59 |
| CTD-2306M5.1 | 1.59 |
| BAZ2A | 1.59 |
| AC114808.2 | 1.59 |
| AC103564.7 | 1.59 |
| AC093642.4 | 1.59 |
| AC073071.1 | 1.59 |
| AC004448.5 | 1.59 |
| WLS | 1.58 |
| TRBV6-7 | 1.58 |
| SNORD115-33 | 1.58 |
| RP11-94H18.2 | 1.58 |
| RP11-666A20.4 | 1.58 |
| RP11-350J20.5 | 1.58 |
| RP11-321L2.2 | 1.58 |
| RP11-302I18.3 | 1.58 |
| RNU7-94P | 1.58 |
| RNU6-771P | 1.58 |
| RNU6-649P | 1.58 |
| RNU6-510P | 1.58 |
| RNU6-45P | 1.58 |
| RNU6-1; RNU6-2 | 1.58 |
| RNU6-15P | 1.58 |
| RNU6-1128P | 1.58 |
| RNU6-1072P | 1.58 |
| PER2 | 1.58 |
| OR7E24 | 1.58 |
| LST1 | 1.58 |
| LOC101928205; AC007461.2 | 1.58 |
| CT47A10; CT47A9; CT47A6; CT47A11; CT47A7; CT47A8; CT47A5; CT47A4; CT47A3; CT47A2; CT47A1; CT47A12; RP6-166C19.3 | 1.58 |
| CT47A10; CT47A9; CT47A11; CT47A7; CT47A8; CT47A6; CT47A5; CT47A4; CT47A3; CT47A2; CT47A1; CT47A12; RP6-166C19.9 | 1.58 |
| CT47A10; CT47A9; CT47A11; CT47A7; CT47A8; CT47A6; CT47A5; CT47A4; CT47A3; CT47A2; CT47A1; CT47A12; RP6-166C19.8 | 1.58 |
| CT47A10; CT47A9; CT47A11; CT47A7; CT47A8; CT47A6; CT47A5; CT47A4; CT47A3; CT47A2; CT47A1; CT47A12; RP6-166C19.4 | 1.58 |
| CT47A10; CT47A9; CT47A11; CT47A7; CT47A8; CT47A6; CT47A5; CT47A4; CT47A3; CT47A2; CT47A1; CT47A12; RP6-166C19.2 | 1.58 |
| CT47A10; CT47A9; CT47A11; CT47A7; CT47A8; CT47A6; CT47A5; CT47A4; CT47A3; CT47A2; CT47A1; CT47A12; RP6-166C19.11 | 1.58 |
| CT47A10; CT47A9; CT47A11; CT47A7; CT47A8; CT47A6; CT47A5; CT47A4; CT47A3; CT47A2; CT47A1; CT47A12; RP6-166C19.10 | 1.58 |
| CACHD1 | 1.58 |
| AES; PRR20A; PRR20B; PRR20C; PRR20D; PRR20E | 1.58 |
| AC005808.3 | 1.58 |
| ZNF704 | 1.57 |
| USP9Y | 1.57 |
| SNORD85 | 1.57 |
| SNORD114-27 | 1.57 |
| SAFB | 1.57 |
| RP11-148K1.10 | 1.57 |
| RNU7-10P | 1.57 |
| RNU6-524P | 1.57 |
| RNU6-400P | 1.57 |
| RNU6-33P | 1.57 |
| RNU6-27P | 1.57 |
| RNU6-148P | 1.57 |
| RNU6-1273P | 1.57 |
| RNU6-1172P | 1.57 |
| RNU6-1147P | 1.57 |
| RNU5D-2P | 1.57 |
| RNASE10 | 1.57 |
| PLEKHA2 | 1.57 |
| NOMO3; NOMO2 | 1.57 |
| NOMO2 | 1.57 |
| LTA | 1.57 |
| LOC100507351 | 1.57 |
| KRTAP5-8 | 1.57 |
| KIAA1549L; C11orf41 | 1.57 |
| KCNC4 | 1.57 |
| FAM90A22P | 1.57 |
| DHX33 | 1.57 |
| DDX11L2 | 1.57 |
| ZSCAN23 | 1.56 |
| SPDYE8P | 1.56 |
| RP11-347K2.2 | 1.56 |
| RP11-33G16.1 | 1.56 |
| RNU7-147P | 1.56 |
| RNU7-105P | 1.56 |
| RNU6-694P | 1.56 |
| RNU6-434P | 1.56 |
| RNU6-37P | 1.56 |
| RNU6-30P | 1.56 |
| RNU6-2; RNU6-1; RNU6-9 | 1.56 |
| RNU6-1; RNU6-7; RNU6-8 | 1.56 |
| RNU6-193P | 1.56 |
| RNU6-11P | 1.56 |
| RNU6-1091P | 1.56 |
| RNU2-44P | 1.56 |
| RNF145 | 1.56 |
| RNA5SP59 | 1.56 |
| RNA5SP100 | 1.56 |
| OR7E156P | 1.56 |
| OR4F29; OR4F3; OR4F16 | 1.56 |
| NOMO1 | 1.56 |
| NOLC1 | 1.56 |
| MIR4531 | 1.56 |
| LOC729444; LOC729461; FAM230A; XXbac-B33L19.3 | 1.56 |
| LOC440896; LOC101927424; LOC101929800; RP11-561O23.5 | 1.56 |
| LOC392196 | 1.56 |
| KRTAP4-12 | 1.56 |
| IGKV2-29 | 1.56 |
| HSPA12A; RP11-539I5.1 | 1.56 |
| FAM230C; LINC00281; LOC642633; KB-1183D5.13 | 1.56 |
| CTD-2197I11.1 | 1.56 |
| CTC-436K13.3 | 1.56 |
| CTAGE4; LOC101060696 | 1.56 |
| ARHGEF2; RP11-336K24.4 | 1.56 |
| ARHGAP42 | 1.56 |
| TMEM131 | 1.55 |
| SRCAP | 1.55 |
| RRM1-AS1 | 1.55 |
| RP4-782G3.1 | 1.55 |
| RP11-622O11.4 | 1.55 |
| RP11-308N19.3 | 1.55 |
| RP11-278H7.3 | 1.55 |
| RNU7-21P | 1.55 |
| RNU7-173P | 1.55 |
| RNU6-60P | 1.55 |
| RNU6-553P | 1.55 |
| RNU6-465P | 1.55 |
| RNU6-379P | 1.55 |
| RNU6-338P | 1.55 |
| RNU6-132P | 1.55 |
| RNU6-1071P | 1.55 |
| PARD6G | 1.55 |
| OR6B2 | 1.55 |
| NSG1 | 1.55 |
| NEU3 | 1.55 |
| MYBBP1A | 1.55 |
| MIR4472-2 | 1.55 |
| MIR4454; LINC00678 | 1.55 |
| MIR1256 | 1.55 |
| MARVELD2 | 1.55 |
| KAT6A | 1.55 |
| HLA-DQB2 | 1.55 |
| FAM197Y2; FAM197Y7; FAM197Y5 | 1.55 |
| EGLN3 | 1.55 |
| CTD-2012I17.1 | 1.55 |
| CNTNAP1 | 1.55 |
| CNKSR3 | 1.55 |
| TAF1 | 1.54 |
| SH3BP4 | 1.54 |
| RP3-323P13.2 | 1.54 |
| RP11-799O21.2 | 1.54 |
| RP11-15G16.1 | 1.54 |
| RNU7-171P | 1.54 |
| RNU7-144P | 1.54 |
| RNU6-944P | 1.54 |
| RNU6-735P | 1.54 |
| OR9G1; OR9G9 | 1.54 |
| OR8G2 | 1.54 |
| OR2A12 | 1.54 |
| MIR4669 | 1.54 |
| LOC401410; RP5-842K16.1 | 1.54 |
| LOC101928844 | 1.54 |
| LOC100129461 | 1.54 |
| LDLRAD3 | 1.54 |
| JMY | 1.54 |
| IGHV3-7 | 1.54 |
| CERS6-AS1 | 1.54 |
| AC091199.1 | 1.54 |
| AC002480.4 | 1.54 |
| XPO5 | 1.53 |
| VN1R3 | 1.53 |
| TNXB; ATF6B | 1.53 |
| SYNE2 | 1.53 |
| STMND1 | 1.53 |
| STAT5A | 1.53 |
| SPRR2E | 1.53 |
| SNX27; RP11-404E16.1 | 1.53 |
| SEC16A | 1.53 |
| RP5-845O24.8 | 1.53 |
| RP11-439L18.1 | 1.53 |
| RP11-374M1.2 | 1.53 |
| RP11-346D19.1 | 1.53 |
| RNU7-61P | 1.53 |
| RNU6-847P | 1.53 |
| RNU6-640P | 1.53 |
| RNU6-584P | 1.53 |
| RNU6-419P | 1.53 |
| RNU6-414P | 1.53 |
| RNU6-366P | 1.53 |
| RNU6-353P | 1.53 |
| RNU6-345P | 1.53 |
| RNU6-1323P | 1.53 |
| RNU6-1306P | 1.53 |
| RNU6-1149P | 1.53 |
| RNU6-1050P | 1.53 |
| RNU6-1019P | 1.53 |
| OR4X2 | 1.53 |
| MIR1976 | 1.53 |
| MGC27345 | 1.53 |
| LOC729461; LOC642633; AC011718.2 | 1.53 |
| LOC101928399; AP001046.5 | 1.53 |
| IGKV2D-29 | 1.53 |
| FAM27C; FAM27B | 1.53 |
| DEFB108B | 1.53 |
| DDX24 | 1.53 |
| CTC-498J12.1 | 1.53 |
| AL163953.3 | 1.53 |
| ACACA | 1.53 |
| ZFP92 | 1.52 |
| UCHL1-AS1 | 1.52 |
| RP5-998N21.4 | 1.52 |
| RP13-30A9.1 | 1.52 |
| RP1-231P7P.1 | 1.52 |
| RP11-327I22.4 | 1.52 |
| RP11-274B18.2 | 1.52 |
| RP11-124O11.1 | 1.52 |
| RNU6-957P | 1.52 |
| RNU6-424P | 1.52 |
| RNU6-367P | 1.52 |
| RNU6-35P | 1.52 |
| RNU6-238P | 1.52 |
| RNU6-1251P | 1.52 |
| RNU6-1099P | 1.52 |
| RNU6-1078P | 1.52 |
| RNU4-65P | 1.52 |
| PLAT | 1.52 |
| LRP6 | 1.52 |
| LPCAT1 | 1.52 |
| LIG3 | 1.52 |
| KB-1507C5.3 | 1.52 |
| HAVCR1P1 | 1.52 |
| GSTA4 | 1.52 |
| GLG1 | 1.52 |
| FMN1 | 1.52 |
| DUX4L2; DUX4L4; DUX2; DUX4; DUX4L1; DUX4L8; DUX4L7; DUX4L6; DUX4L5; DUX4L3 | 1.52 |
| DUX2 | 1.52 |
| CTA-407F11.7 | 1.52 |
| CT47A6; CT47A1; CT47A11; CT47A7; CT47A10; CT47A9; CT47A8; CT47A5; CT47A4; CT47A3; CT47A2; CT47A12; RP6-166C19.1 | 1.52 |
| ANKRD44 | 1.52 |
| AMZ2P1 | 1.52 |
| ALG13-AS1 | 1.52 |
| AC079779.5 | 1.52 |
| AC068039.4 | 1.52 |
| SEPT3 | 1.51 |
| RP5-858B6.1 | 1.51 |
| RP11-764K9.1 | 1.51 |
| RP11-597D13.8 | 1.51 |
| RP11-177F11.1 | 1.51 |
| RNU6-755P | 1.51 |
| RNU6-560P | 1.51 |
| RNU6-499P | 1.51 |
| RNU6-476P | 1.51 |
| RNU6-214P | 1.51 |
| RNU6-1218P | 1.51 |
| RNU4-56P | 1.51 |
| OR9A2 | 1.51 |
| OR2T27 | 1.51 |
| MIR3180-4 | 1.51 |
| MIR1271 | 1.51 |
| LOC729739 | 1.51 |
| LOC101928254; RP11-3B12.3 | 1.51 |
| LOC100293211; IGHV3-11 | 1.51 |
| ITGA4 | 1.51 |
| INTS9 | 1.51 |
| IGKV3D-7 | 1.51 |
| IGKV1-6 | 1.51 |
| IGHV3-30 | 1.51 |
| GS1-433O24.1 | 1.51 |
| GAGE1; GAGE4; GAGE5; GAGE6 | 1.51 |
| DNMBP | 1.51 |
| CT47A10; CT47A9; CT47A6; CT47A1; CT47A11; CT47A7; CT47A8; CT47A5; CT47A4; CT47A3; CT47A2; CT47A12; RP6-166C19.5; RP6-166C19.6 | 1.51 |
| COX7A2L | 1.51 |
| CNTN1 | 1.51 |
| CASP2 | 1.51 |
| AF127936.3 | 1.51 |
| AC114813.1 | 1.51 |
| ZNF836 | -1.51 |
| ZNF776 | -1.51 |
| VPS25 | -1.51 |
| UBE2B | -1.51 |
| SRGAP1 | -1.51 |
| SIKE1 | -1.51 |
| SET | -1.51 |
| SERPINE2 | -1.51 |
| RPS15AP10 | -1.51 |
| RNA5SP45 | -1.51 |
| RCHY1 | -1.51 |
| RAP1B | -1.51 |
| PIGC; LOC100505991 | -1.51 |
| NBEAL1 | -1.51 |
| MORC1 | -1.51 |
| MANEA | -1.51 |
| HPRT1 | -1.51 |
| HJURP | -1.51 |
| HDAC9 | -1.51 |
| FEM1C | -1.51 |
| ERVK-7; RP11-398H6.1 | -1.51 |
| ELP2 | -1.51 |
| EFCAB7 | -1.51 |
| DUSP22; LOC100653247 | -1.51 |
| DPY30 | -1.51 |
| DPH3 | -1.51 |
| COPZ1 | -1.51 |
| COG6 | -1.51 |
| CNN2 | -1.51 |
| CCNL1 | -1.51 |
| C1GALT1 | -1.51 |
| BNIP3P1 | -1.51 |
| AUP1 | -1.51 |
| ARPC5 | -1.51 |
| ANG; RNASE4 | -1.51 |
| AIDA | -1.51 |
| AC092755.4 | -1.51 |
| ZNF551 | -1.52 |
| ZNF468 | -1.52 |
| ZNF432 | -1.52 |
| ZCCHC9 | -1.52 |
| WAC-AS1 | -1.52 |
| VAMP7 | -1.52 |
| TMCO3 | -1.52 |
| STAMBPL1 | -1.52 |
| SPTLC3 | -1.52 |
| SOAT1 | -1.52 |
| PROS1 | -1.52 |
| PRKAB2; LOC101060511 | -1.52 |
| PDP1 | -1.52 |
| MXRA7 | -1.52 |
| MRPL49 | -1.52 |
| LYPLAL1 | -1.52 |
| LOC729291 | -1.52 |
| LINC00493 | -1.52 |
| KITLG | -1.52 |
| IFNGR1 | -1.52 |
| HSD17B11 | -1.52 |
| H2AFZ | -1.52 |
| EXOC5 | -1.52 |
| ENO1-IT1 | -1.52 |
| ELL2 | -1.52 |
| DGCR11 | -1.52 |
| CREBZF | -1.52 |
| ATP1B1 | -1.52 |
| AKTIP | -1.52 |
| ACADVL | -1.52 |
| ZNF33B | -1.53 |
| VPS29 | -1.53 |
| TMEM19 | -1.53 |
| STAG1 | -1.53 |
| SMG1P5 | -1.53 |
| SKAP2 | -1.53 |
| RP11-367B6.2 | -1.53 |
| RNU11-6P | -1.53 |
| RASAL2-AS1 | -1.53 |
| PVRL3 | -1.53 |
| PPP2R3C | -1.53 |
| POC1B; POC1B-GALNT4; GALNT4 | -1.53 |
| PIGA | -1.53 |
| PGBD4 | -1.53 |
| ORC3; RP3-486L4.3 | -1.53 |
| NOP10 | -1.53 |
| NINJ1 | -1.53 |
| N4BP2L2 | -1.53 |
| MAP3K2 | -1.53 |
| LYRM5 | -1.53 |
| LOC101927523; RP11-127O4.3 | -1.53 |
| LIN9 | -1.53 |
| KLHL22-IT1 | -1.53 |
| ITGA1; PELO | -1.53 |
| INSL4 | -1.53 |
| IER3IP1 | -1.53 |
| FAM214A | -1.53 |
| DPY19L1P1 | -1.53 |
| COMMD3 | -1.53 |
| CDK2AP1 | -1.53 |
| CCDC62 | -1.53 |
| CCDC167 | -1.53 |
| BACE2 | -1.53 |
| ATG5 | -1.53 |
| ZNF699 | -1.54 |
| ZNF256 | -1.54 |
| ZDHHC13 | -1.54 |
| SMDT1 | -1.54 |
| RNMT | -1.54 |
| RNF149; SNORD89 | -1.54 |
| RNA5SP82 | -1.54 |
| RNA5SP449 | -1.54 |
| MR1 | -1.54 |
| MIR644A | -1.54 |
| MIR3136 | -1.54 |
| METTL18 | -1.54 |
| MAD2L1 | -1.54 |
| LYPLA1 | -1.54 |
| ISCA1P1 | -1.54 |
| HHAT | -1.54 |
| DDX50 | -1.54 |
| CYFIP2 | -1.54 |
| CAMK2N1 | -1.54 |
| ATP5L | -1.54 |
| ARL8B | -1.54 |
| ARID5B | -1.54 |
| ARHGEF28 | -1.54 |
| AIM2 | -1.54 |
| ZBTB8A | -1.55 |
| UBE2D2 | -1.55 |
| TLR6 | -1.55 |
| SFXN3 | -1.55 |
| RP11-159G9.5 | -1.55 |
| RNU6-638P | -1.55 |
| PSMA6 | -1.55 |
| PCNP | -1.55 |
| PCGF5 | -1.55 |
| NSL1 | -1.55 |
| NDFIP1 | -1.55 |
| MPC1; BRP44L | -1.55 |
| MNAT1 | -1.55 |
| MIR4742 | -1.55 |
| MGC24103 | -1.55 |
| METTL5 | -1.55 |
| MAPK4 | -1.55 |
| HCCS | -1.55 |
| GNPDA2 | -1.55 |
| FUNDC2 | -1.55 |
| FAM104B | -1.55 |
| DTWD1 | -1.55 |
| DCLRE1A | -1.55 |
| CSTB | -1.55 |
| CRBN | -1.55 |
| CGGBP1 | -1.55 |
| CCDC82 | -1.55 |
| CCDC18 | -1.55 |
| CAPN2 | -1.55 |
| BNIP3 | -1.55 |
| BLOC1S1 | -1.55 |
| ATP6V0E2 | -1.55 |
| ARFGAP3; PACSIN2 | -1.55 |
| AHR | -1.55 |
| SPRY1 | -1.56 |
| SLC7A5P2 | -1.56 |
| SKA2 | -1.56 |
| RPSAP52 | -1.56 |
| RNA5SP152 | -1.56 |
| ORMDL1 | -1.56 |
| MTMR6 | -1.56 |
| MMP14 | -1.56 |
| LSM5 | -1.56 |
| LOC284513 | -1.56 |
| LIPT2 | -1.56 |
| IPMK | -1.56 |
| HPGD | -1.56 |
| HIGD1A | -1.56 |
| FRG1; LOC100289097; LOC101930278; LOC101930531 | -1.56 |
| FLOT1 | -1.56 |
| EBLN2 | -1.56 |
| DNAJB6 | -1.56 |
| DHPS | -1.56 |
| CENPW | -1.56 |
| CAAP1 | -1.56 |
| C9orf72 | -1.56 |
| C6orf211 | -1.56 |
| TMEM5 | -1.57 |
| TIMM8B | -1.57 |
| THOC7 | -1.57 |
| TGFBI; LOC100652886; LOC100653157 | -1.57 |
| TBCE; RP11-293G6__A.2 | -1.57 |
| TANK | -1.57 |
| SEMA3A | -1.57 |
| RP11-6N13.1 | -1.57 |
| RNY5P1 | -1.57 |
| RNA5SP129 | -1.57 |
| REXO2 | -1.57 |
| PTPN12 | -1.57 |
| ORMDL2 | -1.57 |
| NCAPG | -1.57 |
| MASTL | -1.57 |
| LMBRD2 | -1.57 |
| LINC-PINT | -1.57 |
| LINC01293; AC104135.4 | -1.57 |
| LAMP2 | -1.57 |
| KIF18A | -1.57 |
| KCTD7; RABGEF1 | -1.57 |
| IKBIP | -1.57 |
| HSBP1L1 | -1.57 |
| GOLGA8B; GOLGA8A; LOC100508892; LOC101930583 | -1.57 |
| GOLGA8A | -1.57 |
| FAM210A | -1.57 |
| FAM204A | -1.57 |
| FAM111A | -1.57 |
| CKLF; CKLF-CMTM1 | -1.57 |
| CENPJ | -1.57 |
| CAPZA1 | -1.57 |
| ARHGEF35 | -1.57 |
| ACBD5 | -1.57 |
| ZNF638; ZNF638-IT1 | -1.58 |
| TXNL1 | -1.58 |
| STYX | -1.58 |
| STXBP3 | -1.58 |
| STAG3L4 | -1.58 |
| SNORD78 | -1.58 |
| SNORA47 | -1.58 |
| RP4-798A10.4 | -1.58 |
| PXMP2 | -1.58 |
| LANCL1 | -1.58 |
| KNTC1 | -1.58 |
| INSIG2 | -1.58 |
| IMMP1L | -1.58 |
| EXOC6 | -1.58 |
| DSN1 | -1.58 |
| DNAJC3 | -1.58 |
| CMTM3 | -1.58 |
| CMC1 | -1.58 |
| CHRNA5 | -1.58 |
| CHMP5 | -1.58 |
| C3orf58 | -1.58 |
| ATP6V1C1 | -1.58 |
| ACTR10 | -1.58 |
| XRCC6BP1 | -1.59 |
| WEE1 | -1.59 |
| WDHD1 | -1.59 |
| UCHL3 | -1.59 |
| TRIM52 | -1.59 |
| SRSF1 | -1.59 |
| SNORD11 | -1.59 |
| SNAP23 | -1.59 |
| SLC25A16 | -1.59 |
| RP11-522D2.1 | -1.59 |
| PRDX6 | -1.59 |
| PDCD2 | -1.59 |
| NABP1 | -1.59 |
| MIR1206 | -1.59 |
| MGAT2 | -1.59 |
| KCNN4 | -1.59 |
| FTSJ1 | -1.59 |
| FBXO8 | -1.59 |
| DHFR | -1.59 |
| CRADD | -1.59 |
| CAP2 | -1.59 |
| C12orf4 | -1.59 |
| BLOC1S1; BLOC1S1-RDH5 | -1.59 |
| ZNF654 | -1.6 |
| VRK1 | -1.6 |
| TUBE1 | -1.6 |
| TRAPPC13; CTC-534A2.2 | -1.6 |
| TRAM1 | -1.6 |
| TIPIN | -1.6 |
| SYS1-DBNDD2 | -1.6 |
| STAM2 | -1.6 |
| SPPL2A | -1.6 |
| SH3BP5-AS1 | -1.6 |
| RP11-272P10.2 | -1.6 |
| RNU1-85P | -1.6 |
| NFU1 | -1.6 |
| MRPL46 | -1.6 |
| LPCAT2 | -1.6 |
| LMBRD1 | -1.6 |
| IFRD1 | -1.6 |
| FTX | -1.6 |
| EVI2B | -1.6 |
| ECT2 | -1.6 |
| CTSO | -1.6 |
| CHAC2 | -1.6 |
| ABCD3 | -1.6 |
| ZNF562 | -1.61 |
| VMA21 | -1.61 |
| TPM1 | -1.61 |
| SLC9A6 | -1.61 |
| SLC19A2 | -1.61 |
| SBF2-AS1 | -1.61 |
| RNA5SP294 | -1.61 |
| RBBP8; MIR4741 | -1.61 |
| RALGPS2 | -1.61 |
| PHKB | -1.61 |
| NOP58 | -1.61 |
| INPP4B | -1.61 |
| FERMT2 | -1.61 |
| DUT | -1.61 |
| DSP | -1.61 |
| DDX3Y | -1.61 |
| CHMP2B | -1.61 |
| CENPU; MLF1IP | -1.61 |
| ATOX1 | -1.61 |
| ACAD11 | -1.61 |
| USP1 | -1.62 |
| TRAPPC2P1 | -1.62 |
| SFT2D3 | -1.62 |
| RNY1P7 | -1.62 |
| PDZD11 | -1.62 |
| MIR17HG; MIR17; MIR18A; MIR19A; MIR19B1; MIR20A; MIR92A1; MIR18B | -1.62 |
| LY75 | -1.62 |
| KAT2B | -1.62 |
| GPRC5A; MIR614 | -1.62 |
| ERLEC1 | -1.62 |
| DLEU2; MIR15A; LINC00371 | -1.62 |
| CRNDE | -1.62 |
| COX20 | -1.62 |
| CORO1C | -1.62 |
| WDR47 | -1.63 |
| UBE2W | -1.63 |
| TMEFF1 | -1.63 |
| STX2 | -1.63 |
| SMYD2 | -1.63 |
| RAB3IP | -1.63 |
| PI4K2B; SEPSECS-AS1; LOC285540 | -1.63 |
| NDRG1 | -1.63 |
| LOC727916 | -1.63 |
| LINC00294 | -1.63 |
| DUSP1 | -1.63 |
| CCNDBP1 | -1.63 |
| BCAP29 | -1.63 |
| TMEM8A | -1.64 |
| TMEM65 | -1.64 |
| STK17A | -1.64 |
| SIPA1L1 | -1.64 |
| SCARNA21 | -1.64 |
| RRAS | -1.64 |
| PTS | -1.64 |
| NPHP3 | -1.64 |
| MYL6 | -1.64 |
| LINC00888 | -1.64 |
| JKAMP | -1.64 |
| GSTCD | -1.64 |
| FUNDC1 | -1.64 |
| DPM1 | -1.64 |
| CCDC53 | -1.64 |
| ARMC1 | -1.64 |
| ANKRD49 | -1.64 |
| ACTR6 | -1.64 |
| ZNF528 | -1.65 |
| SNX13 | -1.65 |
| RAB18 | -1.65 |
| MIR22HG; MIR22 | -1.65 |
| H19 | -1.65 |
| FLRT2; LOC100506718 | -1.65 |
| CTBS | -1.65 |
| C11orf80 | -1.65 |
| AGAP2-AS1 | -1.65 |
| ZNF252P | -1.66 |
| UBA6 | -1.66 |
| TMEM14C | -1.66 |
| SLC36A4 | -1.66 |
| SFRP2 | -1.66 |
| PUS7L | -1.66 |
| LONRF3 | -1.66 |
| GRAMD1A | -1.66 |
| FSBP | -1.66 |
| CENPH | -1.66 |
| CAMK4 | -1.66 |
| ANKRD13D | -1.66 |
| ZFAND2A | -1.67 |
| TMCO1 | -1.67 |
| TIGD4 | -1.67 |
| SYNC | -1.67 |
| SBDSP1 | -1.67 |
| RP11-284F21.7 | -1.67 |
| RP11-274H2.3 | -1.67 |
| RNA5SP81 | -1.67 |
| RNA5SP67 | -1.67 |
| RNA5SP301 | -1.67 |
| RNA5SP267 | -1.67 |
| RNA5SP185 | -1.67 |
| RFWD2 | -1.67 |
| NES | -1.67 |
| NAPG | -1.67 |
| MYH1 | -1.67 |
| MRPL33 | -1.67 |
| MIR590 | -1.67 |
| LRRC40 | -1.67 |
| LOC100507217 | -1.67 |
| HSPA1B | -1.67 |
| GLRX3 | -1.67 |
| EIF2A | -1.67 |
| DRAM2 | -1.67 |
| DCK | -1.67 |
| CLOCK | -1.67 |
| CCNT2 | -1.67 |
| CCNE2 | -1.67 |
| ZNF367 | -1.68 |
| ZNF134 | -1.68 |
| SNX10 | -1.68 |
| SNAR-B2; SNAR-B1 | -1.68 |
| SMPDL3A | -1.68 |
| RCN2 | -1.68 |
| RAD18 | -1.68 |
| PITPNA-AS1 | -1.68 |
| PIM1 | -1.68 |
| PERP | -1.68 |
| NIPSNAP3A | -1.68 |
| NDFIP2 | -1.68 |
| MFN1 | -1.68 |
| LOC101926963; AC147651.4 | -1.68 |
| FCHO2 | -1.68 |
| COQ10B | -1.68 |
| CCNC | -1.68 |
| CCDC14 | -1.68 |
| BICC1 | -1.68 |
| ANAPC16 | -1.68 |
| THUMPD3-AS1; SETD5-AS1 | -1.69 |
| SNAP25 | -1.69 |
| RAD51AP1 | -1.69 |
| MIR17; MIR18A; MIR19A; MIR19B1; MIR20A; MIR92A1; MIR17HG | -1.69 |
| FAM198B | -1.69 |
| FAM188A | -1.69 |
| DCBLD2 | -1.69 |
| CSAG3; CSAG2 | -1.69 |
| C8orf59 | -1.69 |
| C10orf32 | -1.69 |
| BARD1 | -1.69 |
| ZWILCH | -1.7 |
| VRK2 | -1.7 |
| TDP2 | -1.7 |
| RNA5SP345 | -1.7 |
| PLK4 | -1.7 |
| NEDD4 | -1.7 |
| LINC00473 | -1.7 |
| INF2 | -1.7 |
| INADL | -1.7 |
| IFI30 | -1.7 |
| HINT1 | -1.7 |
| ETAA1 | -1.7 |
| EIF2S1 | -1.7 |
| CENPL | -1.7 |
| ZNF772 | -1.71 |
| USP16 | -1.71 |
| UQCRB | -1.71 |
| TTC37 | -1.71 |
| TOMM5 | -1.71 |
| PPP1CB | -1.71 |
| PCDHB16 | -1.71 |
| PAWR | -1.71 |
| MYH4 | -1.71 |
| MAGT1 | -1.71 |
| LPXN | -1.71 |
| LGALS3 | -1.71 |
| IMMP2L-IT1 | -1.71 |
| HTATIP2 | -1.71 |
| EBAG9 | -1.71 |
| SLC26A2 | -1.72 |
| SAP30 | -1.72 |
| RNU7-189P | -1.72 |
| PLP2 | -1.72 |
| NT5DC2 | -1.72 |
| GBP3 | -1.72 |
| FSD1L | -1.72 |
| FAM20B | -1.72 |
| DRAM1 | -1.72 |
| CD46 | -1.72 |
| CASP8AP2 | -1.72 |
| BIRC3 | -1.72 |
| BET1 | -1.72 |
| UBA3 | -1.73 |
| TFPI2 | -1.73 |
| RNA5SP86 | -1.73 |
| NFIL3 | -1.73 |
| MCM8 | -1.73 |
| LOC644656 | -1.73 |
| FAM102A | -1.73 |
| ERI2 | -1.73 |
| CHL1 | -1.73 |
| AC104655.3 | -1.73 |
| AC017002.2 | -1.73 |
| ZNF827 | -1.74 |
| STX6; RP11-46A10.5 | -1.74 |
| SNORD101; SNORD100; SNORA33 | -1.74 |
| RNY5P8 | -1.74 |
| RNY5 | -1.74 |
| RBL1 | -1.74 |
| PCNA | -1.74 |
| NUF2 | -1.74 |
| MIR1278 | -1.74 |
| MED21; LOC101928625 | -1.74 |
| LSM8; NAA38 | -1.74 |
| LOC284454 | -1.74 |
| HSPB11 | -1.74 |
| GTF2F2 | -1.74 |
| GPN1 | -1.74 |
| GNPAT | -1.74 |
| GBE1 | -1.74 |
| FBXW7 | -1.74 |
| FAM218A | -1.74 |
| DLEU2; MIR15A; MIR16-1 | -1.74 |
| CCDC144CP | -1.74 |
| ZNF511 | -1.75 |
| WIPF1 | -1.75 |
| UHRF2 | -1.75 |
| TOR1AIP2 | -1.75 |
| TMEM167A | -1.75 |
| TBC1D23 | -1.75 |
| SLC25A40 | -1.75 |
| RP11-735G4.1 | -1.75 |
| RNF182 | -1.75 |
| RB1CC1 | -1.75 |
| PEA15 | -1.75 |
| PCMTD1 | -1.75 |
| MYNN | -1.75 |
| KLHDC2 | -1.75 |
| DCTN6 | -1.75 |
| ZNF14 | -1.76 |
| TRIM61 | -1.76 |
| TMEM135 | -1.76 |
| PRKRA | -1.76 |
| NCS1 | -1.76 |
| IPO7 | -1.76 |
| CYLD | -1.76 |
| CENPE | -1.76 |
| C1orf27 | -1.76 |
| TMEM14A | -1.77 |
| SUCO | -1.77 |
| SLC30A9 | -1.77 |
| SARAF; TMEM66 | -1.77 |
| RYK | -1.77 |
| MYL6P1 | -1.77 |
| CYB5R4 | -1.77 |
| ATP6V1G1 | -1.77 |
| ANKRD36B; ANKRD36 | -1.77 |
| TRAM1L1 | -1.78 |
| TMX3 | -1.78 |
| SRSF5 | -1.78 |
| RP11-152P17.2 | -1.78 |
| RASSF8 | -1.78 |
| MOB4; HSPE1-MOB4 | -1.78 |
| LINC01004 | -1.78 |
| KIFAP3 | -1.78 |
| ELOVL7 | -1.78 |
| DDR1 | -1.78 |
| COX17 | -1.78 |
| CAST | -1.78 |
| C1orf112 | -1.78 |
| AP000695.4 | -1.78 |
| YEATS4 | -1.79 |
| TSEN15 | -1.79 |
| SRD5A3 | -1.79 |
| PDLIM5 | -1.79 |
| P4HA1 | -1.79 |
| LOC100294145 | -1.79 |
| ITGB3 | -1.79 |
| COPS8 | -1.79 |
| ATP6V0E1 | -1.79 |
| STC1 | -1.8 |
| RP11-3L8.3 | -1.8 |
| NPHP3-ACAD11 | -1.8 |
| LPP-AS2 | -1.8 |
| LOC642236 | -1.8 |
| LOC389831 | -1.8 |
| LINC01139; RP11-371I1.2 | -1.8 |
| FBXL5 | -1.8 |
| CCDC90B | -1.8 |
| UAP1 | -1.81 |
| SLC25A24 | -1.81 |
| RP11-556E13.1 | -1.81 |
| RNFT1 | -1.81 |
| RNF7 | -1.81 |
| RNA5SP197 | -1.81 |
| PNPLA8 | -1.81 |
| LOC101927841; AC005682.5 | -1.81 |
| ITGB1 | -1.81 |
| HNRNPR | -1.81 |
| GGH | -1.81 |
| C12orf49 | -1.81 |
| ARL6IP5 | -1.81 |
| XPR1 | -1.82 |
| RP4-644L1.2 | -1.82 |
| RIPK2 | -1.82 |
| RAB22A | -1.82 |
| NTAN1 | -1.82 |
| MIR4534 | -1.82 |
| GPN3 | -1.82 |
| DCUN1D1 | -1.82 |
| ATP6V0A4 | -1.82 |
| ZNF280D; LOC145783 | -1.83 |
| UFM1 | -1.83 |
| SCARNA12 | -1.83 |
| RP11-370K11.1 | -1.83 |
| PTPLA | -1.83 |
| GLIPR1 | -1.83 |
| DLGAP1-AS1 | -1.83 |
| C19orf48; SNORD88C | -1.83 |
| ANKRD36C; LOC100996862 | -1.83 |
| ANKRD28 | -1.83 |
| SLC41A2 | -1.84 |
| N4BP2L2-IT2 | -1.84 |
| KCTD9 | -1.84 |
| HNRNPU-AS1 | -1.84 |
| FAM114A1 | -1.84 |
| DKFZP434I0714 | -1.84 |
| CREG1 | -1.84 |
| CAPZA2 | -1.84 |
| ATP6V0B | -1.84 |
| TSPAN17 | -1.85 |
| TSPAN13 | -1.85 |
| RPL37 | -1.85 |
| RP11-492I21.1 | -1.85 |
| DUSP12 | -1.85 |
| CYR61 | -1.85 |
| AMER1 | -1.85 |
| TCTE3 | -1.86 |
| SAT1 | -1.86 |
| PDLIM3 | -1.86 |
| OTUD6B-AS1; GS1-251I9.4 | -1.86 |
| DNAJC19 | -1.86 |
| COMMD8 | -1.86 |
| CHPT1 | -1.86 |
| BROX | -1.86 |
| AGPAT9 | -1.86 |
| RP11-328D5.1 | -1.87 |
| MAPK6 | -1.87 |
| ITGB3BP | -1.87 |
| DYX1C1-CCPG1 | -1.87 |
| DEPDC1 | -1.87 |
| ARL4A | -1.87 |
| ADAM9 | -1.87 |
| VPS8; LOC100505729 | -1.88 |
| USP12 | -1.88 |
| TMEM161B | -1.88 |
| RPL32P3 | -1.88 |
| POC1B | -1.88 |
| MIR3661 | -1.88 |
| LOC101928020; AC007038.7 | -1.88 |
| LINC00645 | -1.88 |
| ANKRD36; ANKRD36C; ANKRD36B | -1.88 |
| AC006372.6 | -1.88 |
| TMEM208 | -1.89 |
| SWT1 | -1.89 |
| PMAIP1 | -1.89 |
| PIGP | -1.89 |
| NR2F1 | -1.89 |
| LOC154761 | -1.89 |
| ETV1 | -1.89 |
| DSCC1 | -1.89 |
| CISD2 | -1.89 |
| SNX7 | -1.9 |
| SNORD12 | -1.9 |
| RSPH3 | -1.9 |
| LOC101928054 | -1.9 |
| LINC01355 | -1.9 |
| CCL20 | -1.9 |
| C8orf37 | -1.9 |
| AP1S2 | -1.9 |
| AGPS | -1.9 |
| AC073218.2 | -1.9 |
| ZNF638-IT1 | -1.91 |
| TIFA | -1.91 |
| OTUD4 | -1.91 |
| CTNNAL1 | -1.91 |
| CNIH4 | -1.91 |
| ATF6 | -1.91 |
| SGTB | -1.92 |
| PET112 | -1.92 |
| MRPL13 | -1.92 |
| MOCS2 | -1.92 |
| GLRX2 | -1.92 |
| FKBP3 | -1.92 |
| CMPK1 | -1.92 |
| CDKN3 | -1.92 |
| TXNDC9 | -1.93 |
| TMSB4X | -1.93 |
| MIR181A2HG | -1.93 |
| KCNH1 | -1.93 |
| HIF1A-AS1 | -1.93 |
| COX7C; MIR3607 | -1.93 |
| ANKRD36C | -1.93 |
| USMG5; MIR1307 | -1.94 |
| TAGLN2 | -1.94 |
| SRP9 | -1.94 |
| SLC39A8 | -1.94 |
| RNA5SP217 | -1.94 |
| RDX | -1.94 |
| NDUFAF2 | -1.94 |
| MARCH7 | -1.94 |
| SNORD14B | -1.95 |
| MRPS14 | -1.95 |
| MMGT1 | -1.95 |
| FAM111B | -1.95 |
| WSB2 | -1.96 |
| TAS2R30 | -1.96 |
| SLC38A6 | -1.96 |
| RAPGEF2 | -1.96 |
| PRMT9; PRMT10 | -1.96 |
| PCNA-AS1 | -1.96 |
| NDUFC1 | -1.96 |
| LGALS1 | -1.96 |
| HAT1 | -1.96 |
| DCAF6 | -1.96 |
| ARSK | -1.96 |
| VTRNA1-2 | -1.97 |
| NFE2L3 | -1.97 |
| JADE1; PHF17 | -1.97 |
| IMPAD1 | -1.97 |
| FURIN | -1.97 |
| CCPG1; MIR628 | -1.97 |
| SLC25A37; FP15737 | -1.98 |
| SELT; RP11-392O18.1 | -1.98 |
| RNU4-9P | -1.98 |
| RNA5SP33 | -1.98 |
| MIR5047 | -1.98 |
| LYPD1 | -1.98 |
| LY6G6E | -1.98 |
| HMMR | -1.98 |
| GORAB | -1.98 |
| CPEB4 | -1.98 |
| AHSA2 | -1.98 |
| SEC11C | -1.99 |
| RASEF | -1.99 |
| MIR221 | -1.99 |
| MIR18B | -1.99 |
| KCNQ1OT1 | -1.99 |
| ETHE1 | -1.99 |
| ERGIC2 | -1.99 |
| CXADR | -1.99 |
| CLK1 | -1.99 |
| ANKRD10 | -1.99 |
| UHMK1 | -2 |
| RAB33B | -2 |
| PLAUR | -2 |
| MURC | -2 |
| GAS5; SNORD74; SNORD47; SNORD44; SNORD81; SNORD79; SNORD80; SNORD76; SNORD77 | -2 |
| TTC39C | -2.01 |
| RPE | -2.01 |
| RNA5SP383 | -2.01 |
| C1orf53 | -2.01 |
| ACAP2-IT1 | -2.01 |
| UGT2B7 | -2.02 |
| SNX14 | -2.02 |
| CUL4A | -2.02 |
| SH3RF1 | -2.03 |
| RNU2-63P | -2.03 |
| PANK2 | -2.03 |
| GXYLT2 | -2.03 |
| CBR4 | -2.03 |
| ARL4C | -2.03 |
| ZBTB38 | -2.04 |
| MMD | -2.04 |
| MIR573 | -2.04 |
| CALM2; CALM1; CALM3 | -2.04 |
| MIR591 | -2.05 |
| MCOLN3 | -2.05 |
| CENPK | -2.05 |
| BLZF1 | -2.05 |
| ATP5L2 | -2.05 |
| GPCPD1 | -2.06 |
| CMC2 | -2.06 |
| CLEC2D | -2.06 |
| CCDC104 | -2.06 |
| TMEM56 | -2.07 |
| RELL1 | -2.07 |
| CLCN3 | -2.07 |
| ANAPC10 | -2.07 |
| TMEM106C | -2.08 |
| SNORD81; SNORD79; SNORD80; SNORD47; SNORD44; GAS5; SNORD74; SNORD76; SNORD77 | -2.09 |
| RNU6-334P | -2.09 |
| FHDC1 | -2.09 |
| AL121578.2 | -2.09 |
| SERPINB8 | -2.1 |
| ETFDH | -2.1 |
| CSGALNACT2 | -2.1 |
| TCEAL1 | -2.11 |
| RP5-826L7.1 | -2.11 |
| RP11-503E24.2 | -2.11 |
| MIR21 | -2.11 |
| LACTB | -2.11 |
| DLGAP1-AS2 | -2.11 |
| SETD7 | -2.12 |
| RNF150 | -2.12 |
| PTPRU | -2.12 |
| HIF1A-AS2 | -2.12 |
| GLRB | -2.12 |
| PPAP2A | -2.13 |
| IMPA1 | -2.13 |
| CTSD | -2.13 |
| TMEM45A | -2.14 |
| RP11-222A11.1 | -2.14 |
| PAEP | -2.14 |
| LMAN1 | -2.14 |
| ACYP1 | -2.14 |
| ZC2HC1A | -2.15 |
| TWF1 | -2.15 |
| STX11 | -2.15 |
| RP11-552M14.1 | -2.15 |
| MIR146A; LOC285628; CTC-231O11.1 | -2.15 |
| TRIM2 | -2.16 |
| CACYBP | -2.16 |
| RPS27L | -2.17 |
| PHYH | -2.17 |
| MIR4653 | -2.18 |
| CSGALNACT1 | -2.18 |
| RP11-269F21.2 | -2.19 |
| KCNQ5-IT1 | -2.19 |
| ELF2 | -2.19 |
| DUSP10 | -2.19 |
| UBASH3B | -2.2 |
| RNU11 | -2.2 |
| PRRG4 | -2.2 |
| CENPQ | -2.2 |
| AHNAK2 | -2.2 |
| AC092168.2 | -2.2 |
| CFL2 | -2.21 |
| SOCS3 | -2.22 |
| DENND1B | -2.22 |
| RNY1P5 | -2.23 |
| RMDN3; FAM82A2 | -2.23 |
| SVIP | -2.24 |
| GCH1 | -2.24 |
| C4orf46 | -2.24 |
| TIAM2; LOC100505519 | -2.25 |
| SCLT1; LOC101927137 | -2.25 |
| STK17B | -2.26 |
| RNU7-163P | -2.26 |
| RAB31 | -2.27 |
| LOC340340; RP5-884M6.1 | -2.27 |
| LOC101928020 | -2.27 |
| IL1RAPL1 | -2.27 |
| CFH | -2.27 |
| PRIM1 | -2.28 |
| LOC100190986 | -2.28 |
| ARMCX1 | -2.29 |
| PIGK | -2.31 |
| C4orf33 | -2.31 |
| MGST3; LOC100505828 | -2.32 |
| CCDC109B | -2.32 |
| LINC00518 | -2.33 |
| TIAM2 | -2.34 |
| SPECC1 | -2.34 |
| SCOC | -2.34 |
| KIAA1217 | -2.34 |
| EYA4 | -2.34 |
| ASAP3 | -2.34 |
| TMA16 | -2.35 |
| C4orf27 | -2.35 |
| MAP7 | -2.36 |
| LOC100130954; RP11-98L5.2 | -2.36 |
| LOC285628 | -2.37 |
| GADD45B | -2.37 |
| TBC1D9 | -2.38 |
| SLC10A7 | -2.39 |
| SDIM1 | -2.39 |
| RNU6-1016P | -2.39 |
| OVAAL; RP11-522D2.1 | -2.39 |
| ZNF354B; RNU1-39P | -2.4 |
| TMEM168 | -2.41 |
| NOV | -2.41 |
| LRBA | -2.41 |
| POLE2 | -2.42 |
| PLA2G4A | -2.42 |
| NEK1 | -2.42 |
| ZNF330 | -2.43 |
| EFNA1 | -2.43 |
| TNFRSF12A | -2.44 |
| SGCD | -2.44 |
| RND3 | -2.44 |
| AC009499.2 | -2.44 |
| SMARCA5 | -2.45 |
| PPID | -2.45 |
| RPS3A; SNORD73A | -2.46 |
| ARFIP1 | -2.46 |
| TM4SF1 | -2.47 |
| EEF1E1 | -2.47 |
| VAMP4 | -2.48 |
| TMEM2 | -2.48 |
| PYROXD1 | -2.48 |
| CSRP2 | -2.48 |
| MFSD12 | -2.49 |
| PMEPA1 | -2.5 |
| MOCOS | -2.5 |
| CD44 | -2.5 |
| ARHGAP10 | -2.5 |
| SNORA71C | -2.51 |
| RGS1 | -2.53 |
| NEAT1; MIR612 | -2.53 |
| IVNS1ABP | -2.53 |
| HIF1A | -2.53 |
| PFDN4 | -2.54 |
| LOC100130954 | -2.54 |
| TMEM192 | -2.56 |
| MPC2 | -2.56 |
| MCTP2 | -2.56 |
| NAA15 | -2.59 |
| MIR3142 | -2.59 |
| LSM6 | -2.59 |
| HSD17B7 | -2.59 |
| AC083843.1 | -2.59 |
| EVI2A | -2.61 |
| TMSB4XP8 | -2.62 |
| MIR3140 | -2.62 |
| CDH2 | -2.62 |
| WDR72 | -2.64 |
| HTR1D | -2.64 |
| SYTL5 | -2.65 |
| SPINK1 | -2.66 |
| MYOF | -2.66 |
| DLEU2L | -2.66 |
| HSD17B7P2 | -2.69 |
| TAF7 | -2.7 |
| ST3GAL4 | -2.7 |
| FZD4 | -2.7 |
| SQRDL | -2.73 |
| SNORA49 | -2.73 |
| FSTL3 | -2.75 |
| ENPP4 | -2.77 |
| RNY1 | -2.78 |
| COLGALT2 | -2.82 |
| SNORD14D | -2.83 |
| MALAT1 | -2.83 |
| CD58 | -2.83 |
| SH3D19 | -2.84 |
| CSAG1; CSAG3 | -2.84 |
| KLHL2 | -2.85 |
| FNIP2 | -2.85 |
| ABCE1 | -2.86 |
| POLR2K | -2.87 |
| RASAL2 | -2.88 |
| AC010967.2 | -2.9 |
| PDGFC | -2.92 |
| TMEM184C | -2.96 |
| CLIP4 | -2.98 |
| PLRG1 | -2.99 |
| SCARNA9; SCARNA9L | -3 |
| LINC00630 | -3.01 |
| LRIG3 | -3.05 |
| ATP6V0D2 | -3.05 |
| DYNLT3 | -3.06 |
| BACE2-IT1 | -3.07 |
| USP38 | -3.09 |
| TMEM38B | -3.1 |
| DEPDC7 | -3.11 |
| ANKRD10-IT1 | -3.14 |
| KATNBL1 | -3.16 |
| RPS3AP47 | -3.17 |
| GNG11 | -3.19 |
| LOC101928716; AC004988.1 | -3.25 |
| LINC00456; RP11-199B17.1 | -3.26 |
| MMP2 | -3.27 |
| FOXF2 | -3.27 |
| LINC-PINT; MIR29A; MIR29B1; AC058791.1 | -3.29 |
| LOC100506444; RP11-317M11.1 | -3.37 |
| RP11-11N9.4 | -3.41 |
| ELMOD2 | -3.43 |
| LUZP4 | -3.52 |
| LOC152225; RP11-221J22.1; RP11-221J22.2 | -3.57 |
| LL0XNC01-237H1.2 | -3.58 |
| SLAMF9 | -3.62 |
| PLOD2 | -3.62 |
| ARHGEF9 | -3.63 |
| MIR29A; MIR29B1 | -3.66 |
| CXCL8 | -3.74 |
| LINC00702 | -3.88 |
| LINC00520 | -3.91 |
| RP11-308N19.1 | -3.92 |
| TROVE2 | -3.93 |
| LOC100288721 | -4.01 |
| ANXA1 | -4.01 |
| LINC00973 | -4.02 |
| CDC73 | -4.02 |
| MSMO1 | -4.12 |
| VDR | -4.21 |
| CDH19 | -4.22 |
| CXCL8; IL8 | -4.25 |
| PPAPDC1A | -4.38 |
| ASPM | -4.44 |
| MND1 | -4.49 |
| PNLIPRP3 | -4.55 |
| UCHL5 | -4.65 |
| ITGB8 | -4.66 |
| CCDC147-AS1 | -4.75 |
| ZBTB41 | -4.83 |
| SPARC | -4.88 |
| MMP8 | -4.91 |
| TGFB1 | -5.06 |
| AXL | -5.15 |
| NEK7 | -5.23 |
| EDIL3 | -5.39 |
| CACNA1E | -5.54 |
| SLC7A11 | -5.91 |
| FDCSP | -7.01 |
| LOC101928555 | -8.01 |
| SPIN4 | -8.82 |
| RGS2 | -8.99 |
| SLAMF7 | -11.25 |
| LINC01278; RP11-357C3.3 | -12.41 |
| RP11-550P17.5 | -21.08 |
| L1CAM | -96.76 |
|  |  |
|  |  |
|  |  |
|  |  |
