# Supplementary material for: Knockdown of L1CAM significantly reduces metastasis in a xenograft model of human melanoma: L1CAM is a potential target for anti-melanoma therapy
Source: PLoS One. 2018 Feb 12;13(2):e0192525. doi: 10.1371/journal.pone.0192525 (PMC5809060; doi:10.1371/journal.pone.0192525)
Supplement: S2 Table — Included are only fold changes at least +/- 1.51; ANOVA and adjusted p-values <0.05. (DOCX) [file pone.0192525.s005.docx]

**Supplemental Table 2:** **Comparison of the gene expression of subcutaneous MV3 tumors in scid mice with L1CAM knockdown (MV3 L1 kd) versus expression of MV3 Luc control (MV3 Luc) tumors. Included are only fold changes at least +/- 1.51; ANOVA and adjusted p-values <0.05.**

| **gene symbol fold change** | |  | |
| --- | --- | --- | --- |
| FAM101B | 4.83 | |  |
| CAMK1D; LOC283070 | 2.34 | |  |
| DACT1 | 2.29 | |  |
| SLC44A2 | 2.24 | |  |
| VAMP7 | 1.9 | |  |
| NOX4 | 1.89 | |  |
| LIFR | 1.81 | |  |
| MAP2K6 | 1.79 | |  |
| QSOX2 | 1.78 | |  |
| ZNF594 | 1.74 | |  |
| POLE2 | 1.72 | |  |
| SNHG16 | 1.72 | |  |
| NEIL3 | 1.69 | |  |
| TFRC | 1.67 | |  |
| KIF24 | 1.64 | |  |
| CCNJ | 1.63 | |  |
| MALL | 1.62 | |  |
| PDE7B | 1.6 | |  |
| PTGR1 | 1.6 | |  |
| C11orf24 | 1.57 | |  |
| TXNDC5 | 1.52 | |  |
| GOSR2 | 1.5 | |  |
| ATF6 | -1.5 | |  |
| SMARCC2 | -1.5 | |  |
| KLF5 | -1.51 | |  |
| ELOVL4 | -1.52 | |  |
| TFAM | -1.52 | |  |
| APLP2 | -1.52 | |  |
| TM7SF3 | -1.52 | |  |
| RABGAP1L | -1.53 | |  |
| CCDC6 | -1.54 | |  |
| VWA8; KIAA0564 | -1.54 | |  |
| CHORDC1 | -1.55 | |  |
| RP11-263K19.6 | -1.56 | |  |
| LNPEP | -1.56 | |  |
| TMBIM4 | -1.57 | |  |
| HEATR1 | -1.58 | |  |
| CCDC6 | -1.59 | |  |
| DAP3 | -1.6 | |  |
| BAZ2A | -1.6 | |  |
| OPHN1 | -1.61 | |  |
| MORF4L1 | -1.61 | |  |
| TMEM50B | -1.62 | |  |
| NBPF3 | -1.63 | |  |
| SPTAN1 | -1.66 | |  |
| PHLDA1 | -1.66 | |  |
| ZNF432 | -1.66 | |  |
| NBPF16; NBPF15 | -1.67 | |  |
| RFWD2 | -1.69 | |  |
| RYR2 | -1.7 | |  |
| NBPF9; NOTCH2NL; RP11-458D21.5 | -1.7 | |  |
| TAF1 | -1.71 | |  |
| HUWE1 | -1.71 | |  |
| MORF4 | -1.74 | |  |
| NBPF16; NBPF8; NBPF15 | -1.75 | |  |
| FLJ23867 | -1.76 | |  |
| ARID5B | -1.76 | |  |
| KLHL20 | -1.77 | |  |
| FAM155A-IT1 | -1.77 | |  |
| NME7 | -1.79 | |  |
| NBPF9; NBPF16; NBPF14; NBPF24; LOC101060202; LOC101060684; NBPF8P; NBPF8; NBPF1; NBPF10; NBPF19; NBPF15; RP11-640M9.2; RP3-377D14.1 | -1.8 | |  |
| NPC1 | -1.81 | |  |
| NBPF1; NBPF12; LOC101059961; HYDIN2; NBPF15; NBPF25P | -1.83 | |  |
| TOR1AIP2 | -1.84 | |  |
| TSEN15 | -1.85 | |  |
| NBPF9; NBPF16; NBPF14; NBPF24; LOC101060202; LOC101060684; NBPF8P; NBPF8; NBPF1; NBPF10; NBPF19; NBPF15; RP11-640M9.2; RP3-377D14.1 | -1.86 | |  |
| PRDM8 | -1.87 | |  |
| ACBD6 | -1.88 | |  |
| LOC101059961; NBPF15; WI2-3658N16.1 | -1.89 | |  |
| NBPF14 | -1.89 | |  |
| NBPF9; NBPF14; NBPF12; NBPF7; LOC101060226; NBPF10; NBPF19; NBPF8; NBPF25P; NBPF20 | -1.89 | |  |
| NBPF9; NBPF10; NBPF14; LOC101059961; LOC101060202; LOC101060362; LOC101060684; LOC100506032; NOTCH2NL; NBPF19; NBPF8; NBPF25P | -1.92 | |  |
| FMN2 | -1.93 | |  |
| ITGA2 | -1.94 | |  |
| SOAT1 | -1.96 | |  |
| SMG7 | -1.96 | |  |
| KIFAP3 | -1.97 | |  |
| PHKA1 | -1.97 | |  |
| CACYBP | -2.01 | |  |
| NFAT5 | -2.02 | |  |
| LAMC1 | -2.08 | |  |
| DARS2 | -2.09 | |  |
| XPR1 | -2.12 | |  |
| KCNQ1OT1 | -2.19 | |  |
| PRDX6 | -2.2 | |  |
| L1CAM | -2.2 | |  |
| ITGA10; LOC101060422; RP11-315I20.3 | -2.21 | |  |
| EDEM3 | -2.24 | |  |
| BAI3 | -2.26 | |  |
| TMEM50B | -2.26 | |  |
| TOR1AIP2 | -2.28 | |  |
| SOD2; LOC100129518 | -2.3 | |  |
| FGD6 | -2.53 | |  |
| ABL2 | -2.58 | |  |
| TNFAIP3 | -2.61 | |  |
| RASAL2 | -2.62 | |  |
| ACSS3 | -2.63 | |  |
| PRRC2C | -2.77 | |  |
| FAM129A | -2.92 | |  |
| TPR; RNU6-1240P | -3.15 | |  |
| GPR126 | -3.3 | |  |
| TPR | -3.36 | |  |
| MALAT1 | -5.77 | |  |
